# Relationship between interpersonal trauma exposure and addictive behaviors: a systematic review

**DOI:** 10.1186/s12888-017-1323-1

**Published:** 2017-05-04

**Authors:** Barna Konkolÿ Thege, Lewis Horwood, Linda Slater, Maria C. Tan, David C. Hodgins, T. Cameron Wild

**Affiliations:** 1 Department of Psychology, University of Calgary, 2500 University Drive, Calgary, T2N 1N4 Canada; 2 Research and Academics Division, Waypoint Centre for Mental Health Care, 500 Church Street, Penetanguishene, ON L9M 1G3 Canada; 3 Department of Psychiatry, University of Toronto, 250 College Street, Toronto, M5T 1R8 Canada; 4 John W. Scott Health Sciences Library, University of Alberta, 2K312 WMC University of Alberta, Edmonton, T6G 2R7 Canada; 5 Knowledge Resource Service - Abdul Khaliq Library, Alberta Health Services Cross Cancer Institute, 11560 University Avenue, Edmonton, T6G 1Z2 Canada; 6 School of Public Health, University of Alberta, 3-300 Edmonton Clinic Health Academy, 11405-97 Avenue, Edmonton, T6G 1C9 Canada

**Keywords:** Interpersonal trauma, Posttraumatic stress disorder, Child maltreatment, Substance abuse, Behavioral addiction, Systematic review

## Abstract

**Background:**

The aim of this study was to systematically summarize knowledge on the association between exposure to interpersonal trauma and addictive behaviors. Extant reviews on this association focused on a restricted range of substance-related addictions, and/or used a narrative instead of a systematic approach.

**Methods:**

Systematic searches of 8 databases yielded 29,841 studies, of which 3054 studies were included and subsequently classified in relation to study design (scoping review). A subset of observational studies (*N* = 181) prospectively investigating the relationship between exposure to interpersonal traumata and subsequent behavioral or substance-related addiction problems were characterized. Heterogeneity in study methodologies and types of addictive behaviors and traumatic experiences assessed precluded meta-analysis. Instead, the proportions of associations tested in this literature that revealed positive, negative, or null relationships between trauma exposure and subsequent addictive behaviors were recorded, along with other methodological features.

**Results:**

Of 3054 included studies, 70.7% (*n* = 2160) used a cross-sectional design. In the 181 prospective observational studies (407,041 participants, 98.8% recruited from developed countries), 35.1% of the tested associations between trauma exposure and later addictive behaviors was positive, 1.3% was negative, and 63.6% was non-significant. These results were primarily obtained among non-treatment seeking samples (80.7% of studies; *n* = 146), using single and multi-item measures of addictive behaviors of unknown psychometric quality (46.4% of studies). Positive associations were more frequently observed in studies examining childhood versus adult traumatization (39.7% vs. 29.7%).

**Conclusions:**

Longitudinal research in this area emphasizes alcohol abuse, and almost no research has examined behavioral addictions. Results provide some support for a positive association between exposure to interpersonal trauma and subsequent addictive behaviors but this relationship was not consistently reported. Longitudinal studies typically assessed trauma exposure retrospectively, often after addictive behavior onset, thus precluding robust inferences about whether traumatization affects initial onset of addictive behaviors.

**Electronic supplementary material:**

The online version of this article (doi:10.1186/s12888-017-1323-1) contains supplementary material, which is available to authorized users.

## Background

Addictive disorders are a major public health concern, given their high population prevalence and their associated negative health, social, and economic consequences [1]. The significance of addictions may increase in the future because of recent changes in psychiatric diagnoses and in public opinion, both of which have broadened expert and lay conceptions beyond alcohol and other drugs to include a wide variety of potentially problematic behaviors (e.g., gambling, excessive sexual behavior, overwork, overeating [2, 3]).

Among other genetic, personality, and psychosocial risk factors, psychological trauma is a commonly investigated factor hypothesized to increase vulnerability to the development of addictive disorders [4–8]. Psychological traumata are negative life events or situations that have the potential to cause an extraordinary amount of stress to the individual overwhelming his/her ability to cope and leaving the person in fear of death, annihilation, or insanity. Although studies suggest that individuals experiencing trauma have a greater risk of developing specific addictive disorders than others in the general population [9–11], a broad perspective on the state of scholarship in this area has not been provided to date. This is because the relevant literatures are complex (i.e., researchers tend to focus on specific trauma exposures, substances, and/or addictive behaviors of interest, often over different parts of the life-span, sometimes among treated populations) and interdisciplinary (i.e., study methods are drawn from epidemiology, psychiatry, clinical and social psychology, and victimology, and include both quantitative and qualitative approaches). To our knowledge, this diversity has not yet been systematically characterized. Moreover, extant reviews focus on a small number of substance-related addictions, specific age groups or sexes, and/or use a narrative rather than a systematic approach [5, 8, 12–15]. A systematic overview of the methods and empirical findings in this area is also needed because of the large amount of data accumulated and in light of inconsistent findings observed across studies [1, 16–19].

To address these issues, we conducted a systematic scoping review [20, 21] of the literature on associations between exposure to trauma and addictive behaviors in which a deliberately broad frame was used to include a range of interpersonal traumata and a wide variety of substance-related and behavioral addiction outcomes. As our goal was to provide a comprehensive overview of the literature, we considered both the potentially traumatic events– (e.g., report of childhood sexual abuse) and the trauma-related psychological symptoms (e.g., posttraumatic symptoms after a physical assault) conceptualizations of traumatization. Our rationale for focusing on *interpersonal* traumata was that these represent potentially modifiable risk factors for addictive behaviors, as opposed to non-modifiable risk factors (e.g., natural disasters).

Scoping reviews aim to map rapidly the key concepts underpinning a research area, and the volume, main sources and types of evidence available [22]. Scoping reviews are often conducted before full syntheses and data aggregation (e.g., meta-analysis) when the relevant literature is vast and diverse. A scoping review is particularly appropriate for reviewing the literature on the association between exposure to interpersonal trauma and addictive behaviors because this area is interdisciplinary, conceptually complex, and has not been reviewed comprehensively before. Our specific objectives were to (1) systematically identify and characterize the types of human studies conducted on this association, (2) select longitudinal observational studies from this literature to examine whether this body of higher-quality empirical research supports the idea that an association exists between exposure to traumatic interpersonal events and subsequent addictive behavior, (3) provide a broad overview on the methodologies used in prospective observational studies and to suggest guidelines to improve the quality of scholarship in this area, and (4) identify knowledge gaps and directions for future research.

## Methods

### Search strategy

Eight databases were searched: EBSCO CINAHL Plus with Full-Text, 1937-Current, Ovid EMBASE 1974-Current, MEDLINE, MEDLINE In-Process & Other Non-Indexed Citations 1946-Current, ProQuest PILOTS 1871-Current, Ovid PsycINFO 1806-Current, Scopus 1960-Current, and EBSCO Violence and Abuse Abstracts 1984-Current. Searches were completed on June 10, 2014.

Search terms were initially generated by the entire research team, from which a preliminary set of subject headings were identified using the relevant electronic bibliographic databases. Then, the first 10 records of a separate search were scanned for each subject heading for further possible keywords. The resulting list was reviewed, corrected, and completed by the research team (e.g., adding different spelling variations of certain terms). Table 1 presents the final version of the search term list. Guided by this list, two professional librarians (LS, MT), with expertise in conducting systematic literature review searches, developed a search strategy that included controlled vocabulary (where available) and free-text terms representing the major concepts contained in the review topic. Terms were combined as follows: (substance use OR excessive behavior terms) AND (trauma terms) AND (study design filter). Searches were limited to English language publications. No date limits were applied. Database-specific controlled vocabulary used during the search process is available as a supplementary material to this article (Additional file 1).

**Table 1:** List of general search terms to identify relevant studies

Trauma	Adverse experience, Emotional trauma, Maltreatment, Trauma, Traumas, Traumatic event, Traumatic experience Posttraumatic stress disorder, PTSD Abandonment, Adult survivors of child abuse, Adverse childhood experiences, Battered child syndrome, Child abuse, Child maltreatment, Incest, Molestation, Neglect, Parental aggression Domestic violence, Partner abuse, Partner aggression, Partner violence, Spouse abuse Combat experience, Elder abuse, Prisoner abuse Assault, Attack, Physical abuse, Victimization, Violence, Violent crime Forced sex, Rape, Sexual abuse Emotional abuse, Psychological abuse, Psychological aggression, Verbal abuse
Addictive behavior	Addiction, Addiction-related disorders, Addictive behavior, Behavioral addiction, Dependence, Drug addiction, Drug dependency, Drug-seeking behavior, Hallucinogenic drugs, Narcotic drugs, Polydrug abuse, Psychoactive drugs, Psychoactive prescription drugs, Psychostimulant addiction, Psychotropic drugs, Substance abuse, Substance misuse, Substance use, Substance-related disorders Alcohol abuse, Alcohol addiction, Alcohol dependence, Alcohol dependency, Alcohol drinking, Alcohol use, Alcoholism, Alcohol-related disorders, Binge drinking, Drinking behavior, Harmful drinking, Hazardous drinking, Problem drinking Cigarette consumption, Cigarette smoking, Cigarette use, Nicotine addiction, Nicotine dependence, Nicotine use, Smoking, Tobacco addiction, Tobacco smoking, Tobacco use disorder Cannabis addiction, Cannabis dependence, Cannabis use, Marijuana abuse, Marijuana smoking, Marijuana usage, Marijuana use Cocaine addiction, Cocaine-related disorders, Cocaine use, Compulsive cocaine seeking, Crack Heroin dependence, Opioid addiction, Opioid dependence, Opioid-related disorders, Opioid use Amphetamine, Barbiturates, Designer drug use, Ecstasy, Hallucinogen, Hashish, Inhalant, LSD, Metamphetamine, Metylphenidate, PCP, Phencyclidine, Prescription drug misuse, Psychedelic drugs, Stimulants, Tranquilizer Compulsive gambling, Excessive gambling, Gambling, Gambling addiction, Pathological gambling, Problem gambling Buying addiction, Compulsive buying, Compulsive shopping, Shopping addiction Compulsive exercising, Compulsive running, Dependency on exercise, Excessive exercise, Exercise addiction, Exercise dependence, Sport addiction Excessive internet use, Internet abuse, Internet addiction, Internet misuse, Pathological internet use, Pathological technology addictions, Problematic internet use Computer game addiction, Computer gaming, Computer gaming addiction, Dependence on video games, Game addiction, Gaming abuse, Gaming addiction, Internet gaming addiction, Online game addiction, Online gaming addiction, Problem video game play, Problematic video game use, Problematic video gaming, Video game addiction Addiction to pornography, Compulsive pornography use, Cyberporn dependence, Cyber-sex addiction /cybersex addiction, Pornography addiction, Pornography problem, Sex addiction, Sexual addiction, Sexual compulsivity, Sexually addictive behavior, Sexually compulsive behavior Work addiction, Workaholism Binge-eating disorder, Compulsive eating, Food addiction, Problem eating Binge flying, Compulsive hoarding, Compulsive shoplifting, Compulsive tanning, Love addiction, Mobile phone addiction, Obsessive hoarding, Tanning dependence, Television addiction

**Fig. 1:**
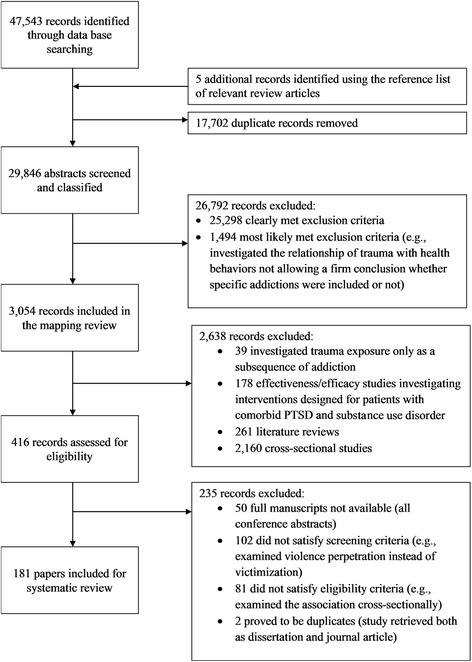
Overview of the study selection process

The database searches retrieved a total of 47,543 references (EBSCO CINAHL Plus – 3513; EMBASE – 11,540; MEDLINE & MEDLINE In-Process & Other Non-Indexed Citations – 8889; ProQuest PILOTS – 5218; PsycInfo – 14,378; Scopus – 2194; Violence and Abuse Abstracts – 1811). After removing duplicates, 29,841 articles remained. Five additional records were identified using the reference lists of relevant review articles; therefore, the total number of records eligible for initial screening was 29,846 (Fig. 1).

### Screening

#### Overview

A three-phased screening process was used. First, we triaged the pool of 29,846 records to identify relevant articles. Second, relevant articles were initially coded to describe study designs in relevant articles (e.g., reviews, cross-sectional studies, and longitudinal studies). Finally, we selected longitudinal studies for detailed coding.

#### Identifying relevant articles: Inclusion and exclusion criteria

Articles were included for further review if they dealt with the relationship between psychological traumata interpersonal in nature and addictive behaviors in human populations. Traumata’ were defined broadly as any *interpersonal* experience (e.g., child abuse, partner violence, bullying, combat experience or the witnessing of these incidents) that has the potential to cause an overwhelming amount of stress to the individual. Addictive behaviors were defined as the use of psychoactive substances (i.e., legal and/or illegal drugs, including misuse of prescription medications) or behaviors either currently classified (i.e., gambling) or under consideration as potential addictions (e.g., sex, video gaming, internet use, overwork, food, and/or shopping). This broad definition included all types of repetitive behaviors that generate short-term rewards despite adverse physical, mental or social consequences, regardless of their current official diagnostic status in psychiatric nosological systems [23–25]. Studies were excluded from further consideration if they defined traumata impersonally (i.e., exposure to natural disasters, accidents or diseases) and/or if they investigated the relationship between traumata and addictive behaviors and a third extrinsic factor without analyzing the relationship of the two main constructs. Studies in languages other than English were also excluded.

#### Initial coding of relevant articles

Relevant records were reviewed and classified into the following five categories: (1) *reviews*, including narrative and systematic reviews, with or without meta-analyses; (2) *cross-sectional studies*, which included qualitative and quantitative studies, as well as studies using mixed qualitative and quantitative designs. Empirical studies were classified as cross-sectional if there was only one assessment point in the study protocol (regardless of how much time passed between the traumatic experience and its assessment in the study). In addition to the review and cross-sectional studies, studies were classified as longitudinal if the study design included at least two assessment points. Longitudinal studies were further divided into three types: (3) *longitudinal intervention studies*, which included studies that investigated the effectiveness or efficacy of interventions designed for individuals with comorbid trauma- and addictive behaviors-related mental health problems, using quantitative, qualitative and/or mixed study designs; (4) *longitudinal studies with trauma as the outcome*, which included studies in which an addictive behavior variable assessed earlier was used to predict a trauma exposure variable assessed later (e.g., effect of alcohol misuse on subsequent sexual victimization), using qualitative, quantitative and/or mixed study designs; and (5) *longitudinal studies with an addictive behavior as the outcome*, which included studies in which a trauma-related variable assessed earlier was used to explain addictive behavior characteristics assessed later (e.g., effect of childhood maltreatment on trajectories of smoking in adulthood), using quantitative, qualitative, and/or mixed study designs. Since addictive behavior outcome was the primary focus of our review, longitudinal studies that assessed both trauma and an addictive behavior as outcomes were included in this last category (versus category 4).

### Detailed coding

Out of the included studies, we selected a subset for detailed coding to examine whether empirical research supports the notion that a longitudinal association between trauma exposure and subsequent addictive behavior exists. This portion of the review focused on best available evidence, that is, prospective observational studies with at least two measurement points where earlier trauma exposure variables were used to predict later addictive behaviors. The total number of studies satisfying these criteria was 181 (See Additional file 2 for detailed bibliographic data).

#### Data extraction

Relevant articles that used longitudinal designs were assessed using a comprehensive coding framework. Two categories of study information were extracted during the coding process: methodological features and study conclusions. As the authors were not aware of any standardized methodological quality assessment tool that covered all study characteristics of interest, methodological features of the studies were evaluated by a custom-made evaluation tool (Table 2). All studies were coded with respect to their analyses of the association between trauma- and addiction-related variables as opposed to the larger study design (e.g., if the trauma-addictive behavior relationship was investigated only in a subsample of the study, then the sample size of this subsample was recorded; similarly, if a study employed several measurement tools to assess addictive behaviors but only one of these was analyzed in relation to trauma exposure, then only this particular measure was coded). If a study could be characterized with several parallel features, more than one code per variable was assigned to the item (e.g., assessing substance misuse using both biological indicators and psychometrically tested questionnaires). We also recorded whether any potential mediators between trauma exposure and addictive disorders were tested in a given study.

**Table 2:** Variables used to describe the methodological characteristics of the studies included in the systematic review

Variable	Codes
Origin of the population studied	USA, Canada, Australia, Western Europe Eastern Europe and Russia Asia (without Russia) Middle and South America Africa
Type of population studied	Non-treatment seekers/general population Non-treatment seekers/student population Non-treatment seekers/other specific population (e.g., medical or military personnel) Treatment seekers/seek treatment for trauma-related mental health issues (e.g., PTSD) Treatment seekers/seek treatment for addiction(s) Treatment seekers/seek treatment for both Treatment seekers/seek treatment for other mental health issue Treatment seekers/seek treatment for other medical reason (e.g., pregnancy)
Sex of the population studied	Male sample Female sample Mixed sample with sex stratification Mixed sample without sex stratification
Sample size	Number of individuals for whom a trauma-addiction relationship was tested
Included target variables at baseline	Assessment included trauma but not addictive behavior Assessment included addictive behavior but not trauma Assessment included both
Included target variables at follow-up(s)	Assessment included trauma but not addictive behavior Assessment included addictive behavior but not trauma Assessment included both Other (e.g., different compositions in different subsequent data waves, no clear description of included variables)
Addictive behaviors included	Alcohol Nicotine Marijuana Cocaine Opiates Prescription medication Other specific substance misuse Substance misuse not specified (e.g., combined measurement of the misuse of several substances) Gambling Shopping Exercise Internet Video games Sex Work Food/eating Other behavioral addiction or combination of several behavioral addictions
Approach to assess addictive behaviors	Continuous measurement (e.g., scale measuring symptom severity) Dichotomous measurement (e.g., nicotine dependence exists or not)
Quality of measurement of the addictive behavior variable(s)	Single item Multi item/ad hoc Multi item/psychometrically tested (with exact name of tool) Biological indicators (e.g., urine testing) Interview or other method
Trauma type studied	Sexual abuse Physical abuse Other/directed towards the individual (e.g., emotional abuse, neglect, bullying) Other/not directed towards the individual (e.g., witnessing serious violence, terror attack) Any specific combination of the above Cannot be determined (e.g., PTSD without any further specification)
Age at trauma onset	Childhood (<18 years) Adult Not specified age
Approach to assess trauma	Continuous measurement of events (e.g., number/frequency of assaults) Dichotomous measurement of events (e.g., rape occurred or not) Continuous measurement of symptoms (e.g., scale measuring PTSD symptom severity) Dichotomous measurement of symptomatology (e.g., PTSD exists or not)
Time interval between trauma exposure and first assessment	In years
Quality of measurement of the trauma variable(s)	Single item Multi item/ad hoc Multi item/psychometrically tested (with exact name of tool) Interview or other method
Dose-response relationship between trauma exposure and addictive symptoms	Dose-response relationship not tested Dose-response relationship studied and found Dose-response relationship tested but not found Dose-response relationship tested but no clear conclusion (e.g., found in one subgroup but not in another)
Mediation between trauma and addiction	Mediators not tested Psychosocial mediators tested Biological mediators tested Both psychosocial and biological mediators tested

Because heterogeneity in study populations, procedures, and measurement of addictive behaviors and traumatic experiences makes meta-analytic procedures quite complex and lengthy, the authors decided not to incorporate them in the present study. Consistent with the goals of a systematic scoping review described earlier, the present paper thus only reports proportions of tested associations revealing positive, negative, or null relationships between trauma exposure and subsequent addictive behaviors. The coding of the results (and thus each four-digit code created) recorded four study characteristics, using the coding categories displayed in Table 2: (1) which type of addictive behavior was investigated; (2) which type of trauma exposure was examined; (3) age at which the trauma occurred; and (4) whether a statistically significant (*p* < 0.05) association was reported with respect to the association being tested (if so, we recorded whether the association was positive or negative; if not, study results were classified as null findings).

Coding of the main conclusions of the studies was conducted on each statistical test reported in the identified subsample analyses, and not at the study level. That is, multiple codes were assigned to the same pair of trauma- addictive behavior variables within the same study if the association was tested by the use of different subsamples (e.g., males versus females, different age groups); assessment tools (e.g., two different instruments assessing alcohol abuse); measurement approaches (e.g., dichotomous versus continuous conceptualization of posttraumatic stress disorder); or indicators of the same construct (e.g., mild versus severe physical abuse).

If an association between a trauma and an addictive behavior was tested by both bivariate and multivariate statistical approaches, only the results of the multivariate analyses were recorded (as these models control for the effects of confounding variables). If both manifest and latent variable approaches were used during the multivariate analyses for the same pair of variables, only the data from the latent variable approach were recorded (as measurement error is smaller in this case). Finally, we also recorded whether a dose-response relationship was reported in a given analysis. Similar to the methodological variables, this was coded at the study rather than the sub-study, observation level.

## Results

### Initial coding

**Fig. 2:**
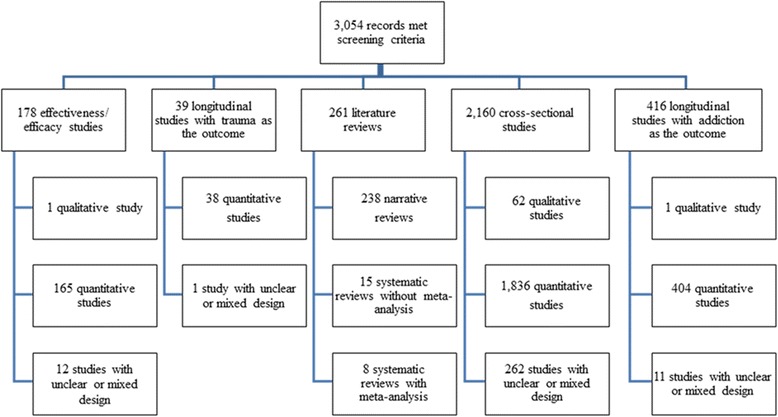
Results of initial coding

The screening of 29,846 titles and abstracts identified 3054 relevant articles that met our review inclusion criteria (Fig. 1). Fully 70.7% (*n* = 2160) of included studies investigated the relationship between exposure to interpersonal trauma and addictive behaviors, using a cross-sectional study design. Longitudinal research was far less common (13.6%; *n* = 416 of included studies), as was intervention efficacy and effectiveness research (5.8%; *n* = 178). Further, of the 261 reviews identified (8.5% of included studies), almost all of them (91.2%; *n* = 238) were narrative and not systematic in nature. Detailed information on the number of investigations per study type is presented in Fig. 2.

### Detailed coding of longitudinal studies

#### Methodological characteristics

Of the 416 longitudinal studies (with the addictive behavior as the outcome) identified during the initial coding, 181 satisfied eligibility criteria (reasons for exclusion in case of 235 records are listed on Fig. 1). The included studies were conducted almost exclusively in the developed Western world (98.8%): only one study (0.6%) was conducted with respondents from Asia and another one (0.6%) with participants from Africa. Concerning population types studied, specific but not treatment-seeking populations (e.g., military personnel) were investigated most often (43.6%) followed by studies conducted in the general population (27.6%). The remaining studies employed samples of (1) individuals seeking treatment for addiction problems (12.7%), (2) students (9.4%), (3) individuals seeking medical care for general health issues (e.g., pregnancy; 3.9%), (4) individuals seeking treatment for mental health issues not related to addictive behaviors and trauma (1.7%), and (5) individuals seeking treatment for trauma-related mental health issues (1.1%). Concerning the sex of the populations studied, most studies (56.4%) used a mixed sample of males and females, 23.8% investigated solely female samples, 16.6% of the studies deployed a mixed sample with sex stratification, while 3.3% of the investigations used solely male samples. Sample sizes ranged between 21 and 48,304 (median = 587) resulting in a total sample size of 407,041 participants for the 181 studies.

With regard to the variables assessed across longitudinal observations, at baseline most studies reported data on both trauma and addictive behavior-related variables (76.8%), while 23.2% assessed only trauma variables. At follow-up(s), again, most studies reported data on both trauma and addictive behavior variables (46.4%), 40.9% included addictive behavior-related variables only, while 12.7% used some other approach (e.g., the scope of included variables varied across survey waves).

Concerning the approach to the measurement of addictive behavior-related constructs, the majority (68.5%) of studies used one variable to assess addictive behaviors (although not necessarily one item; that is, the scale score of a 10-item questionnaire was considered as one variable here) – alcohol misuse being the most common (Table 3). Importantly for the aims of this review, studies of behavioral addictions were almost completely absent: only excessive eating and Internet use were included, each in one study. Addictive behaviors were conceptualized more often as continuous dimensions (49.7%) than dichotomous entities (39.8%); and in a small minority of cases, both approaches were employed within the same study (10.5%). Multi-item, ad hoc questionnaires were used most often to assess addictive behaviors (27.1%), followed by the employment of psychometrically tested measurement instruments (21.0%; names of the specific assessment tools in each study can be found in Additional file 3) and single-item assessments (19.3%). Biological indicators were used only in a small minority of cases (2.2%), while a larger portion (9.9%) of studies employed methods other than those listed above (e.g., interviews) or a combination of them (20.4%).

**Table 3:** Addictive behaviors studied

	N	%
Among studies using one variable to assess addictive behaviors		
Alcohol	57	31.5
Combined measurement of several substances	42	23.2
Nicotine	14	7.7
Marijuana	5	2.8
Cocaine	2	1.1
Other specific substance	2	1.1
Food	1	0.6
Opiates	1	0.6
Among studies using several variables to assess addictive behaviors		
Alcohol + combined measurement of several substances	20	11.0
Alcohol + nicotine	7	3.9
Alcohol + nicotine + combined measurement of several substances	6	3.3
Alcohol + marijuana + combined measurement of several substances	5	2.8
Alcohol + nicotine + marijuana	5	2.8
Alcohol + marijuana + cocaine + opiates + prescription medication	2	1.1
Alcohol + nicotine + marijuana + alcohol + prescription medication	1	0.6
Alcohol + nicotine + marijuana + combined measurement of several substances	1	0.6
Alcohol + marijuana	1	0.6
Alcohol + marijuana + cocaine	1	0.6
Alcohol + marijuana + cocaine + opiates	1	0.6
Alcohol + opiates + marijuana + prescription medication + combined measurement of several substances	1	0.6
Alcohol + prescription medication	1	0.6
Cocaine + opiates	1	0.6
Combined measurement of several substances + excessive Internet use	1	0.6
Marijuana + cocaine + opiates	1	0.6
Marijuana + cocaine + opiates + combined measurement of several substances	1	0.6
Marijuana + cocaine + prescription medication + combined measurement of several substances	1	0.6

Concerning the assessment of interpersonal trauma exposure, the majority (60.8%) of studies used one variable (Table 4). Childhood interpersonal traumas were assessed in 40.9% of the studies, while in 37.0% of the investigations, age at trauma onset was not specified. Adult trauma exposure was investigated in 13.8% of the examinations, and in the remaining 8.3% of studies, populations targeted suffered from traumatic experiences both in childhood and adulthood or in one specific category plus at an undetermined age.

**Table 4:** Traumata studied

	N	%
Among studies using one variable to assess trauma exposure		
Unspecified trauma	40	22.1
Specific combination of trauma types	35	19.3
Sexual abuse^a^	15	8.3
Other (than physical or sexual) abuse directed towards the individual	7	3.9
Other (than physical or sexual) abuse not directed towards the individual	7	3.9
Physical abuse^a^	6	3.3
Among studies using several variables to assess trauma exposure		
Sexual abuse + physical abuse	12	6.6
Specific combination of types + undetermined trauma	10	5.5
Sexual abuse + physical abuse + other abuse directed towards the individual	6	3.3
Other abuse not directed towards the individual + undetermined trauma	6	3.3
Sexual abuse + physical abuse + other abuse directed towards the individual + specific combination of types	5	2.8
Physical abuse + other abuse directed towards the individual	3	1.7
Sexual abuse + physical abuse + other abuse not directed towards the individual	3	1.7
Sexual abuse + other abuse directed towards the individual	3	1.7
Sexual abuse + undetermined trauma	3	1.7
Other abuse directed towards the individual + other abuse not directed towards the individual	2	1.1
Other abuse directed towards the individual + specific combination of types	2	1.1
Physical abuse + specific combination of types	2	1.1
Sexual abuse + physical abuse + other abuse directed towards the individual + other abuse not directed towards the individual	2	1.1
Sexual abuse + physical abuse + other abuse directed towards the individual + other abuse not directed towards the individual + specific combination of types	2	1.1
Sexual abuse + specific combination of types	2	1.1
Other abuse directed towards the individual + undetermined trauma	1	0.6
Other abuse not directed towards the individual + specific combination of types	1	0.6
Physical abuse + other abuse directed towards the individual + specific combination of types	1	0.6
Physical abuse + other abuse not directed towards the individual	1	0.6
Sexual abuse + physical abuse + other abuse not directed towards the individual + specific combination of types	1	0.6
Sexual abuse + physical abuse + other abuse not directed towards the individual + undetermined trauma	1	0.6
Sexual abuse + physical abuse + specific combination of types	1	0.6
Sexual abuse + physical abuse + undetermined trauma	1	0.6

^a^The term abuse is used in the table in a broad sense, including forms of both abuse and assault

Traumatization was typically conceptualized as the occurrence of events and not as the presence of psychological symptoms (Table 5); 40.9% of studies used a dichotomous approach to quantify the extent of trauma exposure, while 30.9% used a continuous measurement approach. The remaining 28.2% of studies approached trauma exposure as the presence of certain trauma-related psychological symptoms or as the combination of the occurrence of events and symptoms. Multi-item questionnaires were used most often to assess trauma exposure; in 37.6% of the studies, these were psychometrically tested instruments (names of the specific assessment tools in each study can be found in Additional file 3), while ad hoc questionnaires were administered in 21.5% of the investigations. Single-item methods were used in 9.4% of the studies to assess trauma exposure and 13.8% employed other methods (e.g., interviews, court documentation). Finally, the remaining 17.7% of the studies employed a combination of these measurement approaches.

**Table 5:** Conceptualization of trauma exposure in the studies

	N	%
Dichotomous measurement of events	74	40.9
Continuous measurement of events	56	30.9
Dichotomous measurement of symptoms	12	6.6
Dichotomous measurement of events + dichotomous measurement of symptoms	9	5.0
Continuous measurement of symptoms	7	3.9
Continuous measurement of events + dichotomous measurement of events	6	3.3
Dichotomous measurement of events + continuous measurement of symptoms	6	3.3
Continuous measurement of events + continuous measurement of symptoms	5	2.8
Continuous measurement of events + dichotomous measurement of symptoms	2	1.1
Continuous measurement of events + dichotomous measurement of events + continuous measurement of symptoms	1	0.6
Continuous measurement of events + dichotomous measurement of events + dichotomous measurement of symptoms	1	0.6
Continuous measurement of symptoms + dichotomous measurement of symptoms	1	0.6
Dichotomous measurement of events + continuous measurement of events	1	0.6

Study authors generally did not quantify the amount of time passed since trauma exposure when collecting the data. An exact time interval was reported only in 1.8% of the studies, while in the remaining 98.2% of studies, this information was not reported (some provided data that allowed the time interval to be approximately estimated – e.g., less than 2 years). Concerning the timing of trauma exposure and first assessment, only a small minority (1.2%) of studies employed a design where the follow-up of an intact population occurred, that is, where the first assessment preceded first trauma exposure. However, even in these cases, the sequence of trauma exposure and addictive behavior onset could not be firmly established (e.g., the follow-up interval was 3 years, within which the order of traumatization and initiation of substance use was not specified).

Concerning the investigation of mediators between interpersonal trauma exposure and addictive disorders, the vast majority (82.2%) of articles did not examine any mediational processes formally. Those that did so typically evaluated psychosocial variables as potential mediators (16.6%), while one study (0.6%) investigated biological mediators and another one (0.6%) included both psychosocial and biological variables.

#### Main conclusion of the studies

The observation-level evaluation of the 181 studies included in our systematic review resulted in 1534 codes. The number of conclusion codes per study ranged between 1 and 54 (median = 6). Almost two-thirds (63.6%) of the tested associations were not significant, contradicting the general assumption in the literature according to which trauma exposure would be an important correlate of addictive behaviors. In more than one third of the cases (35.1%), however, a significant, positive association between the two constructs was reported. Finally, in a very small portion of the observations (1.3%), a significant negative relationship emerged, indicating that trauma exposure may occasionally function as a protective factor against later addictive disorders. Detailed information on the individual trauma- and addictive behavior -level associations between psychological trauma exposure and later addictive disorders is presented in Table 6. Although some individual deviations do occur (e.g., substantially higher proportion of significant positive associations in the case of prescription medication misuse, while a remarkably lower percentage of significant positive associations for opioid use), these subgroup-level data are largely consistent with the main conclusion stated above.

**Table 6:** Individual trauma- and addiction-level description of the associations between trauma exposure and later addiction problems

	Alcohol	Nicotine	Marijuana	Cocaine	Opiates	Prescription medication	Other specific substance misuse	Substance misuse not specified	Internet	Food	Total
Sexual abuse	Σ: 176 0: 70.5% +: 29.5%	Σ: 18 0: 44.4% +: 55.6%	Σ: 22 0: 77.3% +: 22.7%	Σ: 9 0: 66.7% +: 33.3%	Σ:3 0: 66.7% +: 33.3%	N/A	Σ: 2 0: 100%	Σ: 56 0: 73.2% +: 26.8%	N/A	Σ: 2 0: 100%	Σ:288 0: 70.1% +: 29.9%
Physical abuse	Σ: 85 0: 72.9% +: 25.9% -: 1.2%	Σ: 21 0: 42.9% +: 57.1%	Σ: 19 0: 42.1% +: 52.6% -: 5.3%	Σ: 10 0: 90.0% +: 10.0%	Σ: 2 0: 100%	N/A	Σ: 4 0: 100%	Σ: 82 0: 67.1% +: 31.7% -: 1.2	N/A	N/A	Σ: 223 0: 66.8% +: 31.8% -: 1.3%
Other /directed towards the person	Σ: 90 0: 55.6% +: 42.2% -: 2.2	Σ: 14 0: 78.6% +: 21.4%	Σ: 4 0: 75.0% +: 25.0%	Σ: 9 0: 66.7% +: 33.3%	Σ: 1 0: 100%	N/A	Σ: 2 0: 100%	Σ: 34 0: 70.6% +: 29.4%	Σ: 1 +:100%	N/A	Σ: 155 0: 62.6% +: 36.1% -: 1.3%
Other/not directed towards the person	Σ: 63 0: 66.7% +: 33.3%	Σ: 25 0: 72.0% +: 28.0%	Σ: 1 0: 100%	N/A	N/A	Σ: 2 0: 100%	N/A	Σ:25 0: 84.0% +: 16.0%	N/A	N/A	Σ: 116 0: 72.4% +: 27.6%
Specific combination of the above	Σ: 116 0: 56.9% +: 37.1% -: 6.0%	Σ: 38 0: 26.3% +: 73.7%	Σ: 38 0: 50.0% +: 50.0%	Σ:17 0: 76.5% +: 23.5%	Σ: 7 0: 85.7% +: 14.3%	N/A	Σ:5 0: 100%	Σ: 141 0: 55.3% +: 44.7%	N/A	Σ: 2 0: 50.0% +: 50.0%	Σ: 364 0: 54.4% +: 43.7% -: 1.9%
Cannot be determined	Σ: 203 0: 71.4% +: 26.6% -: 2.0%	Σ: 42 0: 50.0% +: 50.0%	Σ: 12 0: 50.0% +: 50.0%	Σ: 18 0: 72.2% +: 27.8%	Σ:13 0: 92.3% +: 7.7%	Σ: 8 0: 25.0% +: 75.0%	Σ: 10 0: 60.0% +: 40.0%	Σ: 82 0: 50.0% +: 45.1% -: 4.9%	N/A	N/A	Σ: 388 0: 63.4% +: 34.5% -: 2.1
Altogether	Σ: 733 0: 66.7% +: 31.4% -: 1.9%	Σ: 158 0: 48.7% +: 51.3%	Σ: 96 0: 56.3% +: 42.7% -: 1.0%	Σ: 63 0: 74.6% +: 25.4%	Σ: 26 0: 88.5% +: 11.5%	Σ: 10 0: 40.0% +: 60.0%	Σ: 23 0: 82.6% +: 17.4%	Σ: 420 0: 61.9% +: 36.9% -: 1.2%	Σ:1 +: 100%	Σ: 4 0: 75.0% +: 25.0%	Σ: 1534 0: 63.6% +: 35.1% -: 1.3%

Note. ^Σ^total number of associations tested; ^0^no association; ^+^positive association; ^−^negative association; ^N/A^no available data

Data on the relationship between trauma exposure and later addictive behavior characteristics as a function of age showed very similar patterns. Specifically, the proportion of tests reporting significant positive associations was 39.7% with trauma exposure in childhood, 29.7% with adult trauma exposure, and 33.0% where age at trauma exposure could not be specified. Rate of significant negative associations between trauma exposure and later addictive behaviors was the highest (2.4%) in studies not specifying the age at trauma exposure, followed by results on adult (1.2%), and childhood (0.6%) traumatization. Trends of the null results also confirmed that the trauma- addictive behavior relationship is stronger when trauma occurs earlier in life: the proportion of non-significant associations was 59.7% with childhood trauma onset, 64.6% in populations suffering from traumatic experiences in an undefined age, while 69.1% with adult trauma exposure.

Dose-response relationship between trauma exposure and subsequent addictive disorders was investigated in 34.3% of the studies. In most of these cases (49.9%), the results were mixed: some of the analyses confirmed, while others disaffirmed the presence of a dose-response relationship between the two constructs. Further, 43.6% of the studies unambiguously supported the assumption that more severe trauma exposure correlated with more severe addictive behavior problems. Finally, a small minority (6.5%) of studies investigated but did not find a dose-response relationship between the two constructs.

## Discussion

### Interpretation of the results

The aim of this systematic review was to summarize and evaluate the empirical data accumulated on the relationship between interpersonal trauma exposure and substance-related and behavioral addictions. The present findings suggest that there is an impoverished evidence base with which to assess this association. Cross-sectional quantitative studies have dominated the literature, and reviews of the area have rarely adopted a systematic approach.

When limiting results of this review only to research that could address whether trauma exposures preceded development of addictive behaviors, i.e., longitudinal studies, about one third of the reviewed observations supported a positive relationship between trauma exposure and addictive behaviors (this ratio was somewhat higher with childhood rather than adult trauma exposure), approximately two thirds did not report a significant association, and a very small minority revealed a significant negative association between the two constructs.

With regard to type of trauma exposure, the highest proportion of significant positive relationships was observed concerning the combined measurement of different potentially traumatizing events, while the lowest ratio of positive relationships emerged concerning adverse experiences not directed towards the specific person (e.g., witnessing violence). With regard to type of addictive behavior, the ranges were substantially wider (11.5–100%); the highest proportion of positive relationships was observed for excessive Internet use (although investigated in only a single study), whereas the lowest rate was found in opiate abuse.

Our results are in line with the findings of two systematic reviews with comparable methodology (i.e., human longitudinal studies examining the relationship between trauma exposure and subsequent development of addictive behaviors). In the work of Devries and colleagues, the authors reported that of 15 longitudinal associations tested between intimate partner violence and subsequent alcohol misuse, six were not significant and two were unclear [5]. Kristman-Valente and Wells similarly reported that almost half of the reviewed longitudinal associations between child maltreatment and substance abuse were not significant in the male or female subsamples they characterized [26]. These reviews of targeted exposures and addictive behaviors are consistent with the present results, i.e., while there is some evidence supporting the relationship between traumatic experiences and addictive behaviors, this association is not always observed, suggesting that future research should investigate factors that might moderate this association (e.g., sex, existence of PTSD over and above trauma exposure, coping strategies and resources).

When interpreting the findings of the present review, one should carefully consider the following factors. First, although we coded and summarized longitudinal studies as part of our systematic review, in the vast majority of these studies, the researchers employed a design where the first assessment occurred long after trauma exposure and often after onset of the addictive behavior as well. As such, the vast majority of human longitudinal studies in this area can be criticized on grounds that their design does not eliminate recall bias [27]. In addition, our results inform whether interpersonal trauma exposure affects the course and not the development of addictive behaviors. Effects of traumatization on the onset of addictive behaviors could ideally be studied by longitudinal studies following populations that are intact in terms of both trauma exposure and addictive behaviors, with assessment points frequent enough to detect the primacy of one of the two phenomena (trauma versus addictive behavior), even if the two occur relatively close to each other in time (as hypothesized). However, the enormous efforts and resources this kind of study design requires explains the absence of this type of investigation in the literature, and verifies the need for this review to summarize findings of relevant studies that used less-than-ideal study designs until higher quality evidence becomes available.

A second aspect worthy of consideration when interpreting the results is the length of the time period within which trauma exposure might affect addictive behaviors. Specifically, it is possible that psychological traumatization may increase vulnerability to addictive behaviors (or their intensification/recurrence) only within a certain time interval (cf. [28]), and for that reason, studies having a long time lag between traumatization and the assessment of addictive behaviors may underestimate the negative effects. This assumption is supported by the evidence present in several studies included in our review, where the misuse of alcohol [18, 29–31], marijuana [32], or a combination of substances [29, 33] was associated with temporally proximal traumatization (typically within a year), but not with earlier trauma history.

This idea, on the other hand, is inconsistent with the fact that the trauma-addictive behavior relationship emerged somewhat more consistently in studies investigating the effects of childhood traumatization in adulthood (that is, typically with a substantially longer time lag). This paradox, however, could be resolved when considering the existence of critical/sensitive periods in the neurobiological processes related to the development of structures and functions of stress regulation. That is, trauma exposure might increase vulnerability to the development of addictive behaviors only in a certain amount of time, unless the adverse experience occurs in a developmentally critical period in which case traumatization could have longer-lasting effects. These considerations call attention to the importance of precisely measuring and reporting the exact timing of adverse events (e.g., exact age) and the time elapsed between trauma exposure and its assessment (and that of addictive disorders) – aspects largely neglected in the literature reviewed here. Similarly, researchers should be more cognizant of the time interval their measurement tools refer to (e.g., lifetime, past month, past year) when attempting to draw robust inferences on the sequence of traumatization and onset of addictive behaviors.

Third, to eliminate as many confounding effects as possible (e.g., the influence of educational level, income, race etc. on addictive behaviors), we gave preference to the results stemming from multivariate analyses; i.e. when results of both bivariate and multivariate investigations were available, data from bivariate analyses were ignored. However, the number and types of covariates in the studies investigated were highly variable across studies and often no conceptual distinction was made between confounding versus explanatory variables. The fact, for instance, that the relationship between trauma exposure and smoking is not significant when controlling for the occurrence of numerous mental disorders as well [34], should not necessarily be understood as the absence of an association; it is plausible that mental health problems (including depression and posttraumatic stress disorder) mediate the association between interpersonal trauma and subsequent addictive behavior [35, 36]. Similarly, the absence of a significant relationship between sexual abuse and alcohol misuse after controlling for distress [37] does not necessarily mean a true absence of an association between traumatization and addictive behaviors. Unfortunately, the methodological trend identified in this literature to conflate confounders and explanatory variables and to treat them simply as ‘covariates’ may also contribute to the large number of null results reported in our review since including a mediator in a model merely as a simple covariate whose effect is controlled in statistical models will obscure mediated associations in statistical tests. It is also worthy of note however, that due to increasing restrictions on manuscript length and the increased sophistication of statistical methods used, increasingly fewer article reports results from bivariate analyses. Therefore, oftentimes only the results of multivariate analyses are available and thus findings of this study would not have been less ambiguous (or would have been substantially less comprehensive) had we decided not to prefer (or include) data from the more complex analyses.

Fourth, our coding system did not distinguish between objective exposure to potentially traumatic events and perceptions/experiences of traumatization among victims. This approach – similar to the objective life events versus perceived stress level dichotomy in psychological stress research [38] – might mistakenly overlook the importance of personal vulnerability, appraisal, and coping resources that can influence whether such exposure in fact leads to psychological traumatization. For example, Chilcoat and Breslau found that posttraumatic stress disorder was associated with an increased risk of drug abuse, while exposure to traumatic events without PTSD did not increase the risk of substance misuse [39]. The absence of the distinction between exposure to potentially traumatic events and actual traumatization in the present work and in the literature in general may also contribute – at least partly – to the high rate of null results found in the present review. It is worthy of mentioning however, that the consistent distinction between trauma exposure and actual psychological traumatization is often neglected in clinical practice as well; therefore, the large proportion of null findings reported in the present work – using the two conceptualizations of trauma combined – might also help clinicians reflect on the importance of making this distinction.

Fifth, low sample sizes in many of the studies may have resulted in insufficient statistical power to detect associations between trauma and subsequent addictive behaviors. In the present review, papers reporting only null results tended (Kruskal-Wallis χ^2^ = 3.6, *p* = 0.057) to have smaller sample sizes (median sample size = 190) than those reporting only positive associations (median sample size = 526) or null and positive associations (median sample size = 751), which supports this possibility. On the other hand, use of very large sample sizes can identify statistically significant associations even in the absence of clinical significance. Thus, future reviews should emphasize effect size estimates as well.

Sixth, consistency and strength of the associations reported may also be subject to recall biases. For instance, a follow-up study investigating the reliability of reports on trauma exposure revealed that 87% of an opioid dependent sample reported at least one traumatic event inconsistently across the two waves of data collection [40]. Later underreporting was exceptionally high (30%) concerning traumatic events that occurred to others (e.g., witnessing violence), which is in line with our results, according to which the absence of a significant relationship between trauma exposure and addictive disorders was most characteristic in the category ‘traumatic event not directed toward the person’. In addition, a recent study showed that while no association emerged between trauma exposure and later alcohol misuse when considering retrospective self-report data on traumatization, the relationship between the two variables proved to be significant in the subsample of respondents who experienced trauma according to ‘real-time’ collateral information but not according to later retrospective self-report [41]. Concerns raised by recall bias issues call our attention to the relevance of animal studies and the importance of prospective investigations using more objective trauma exposure indicators (e.g., court documentation, collateral information).

Finally, even though we aimed to provide a broad, overarching picture on the trauma-addictive behavior relationship, the vast majority of studies reviewed here focused on nicotine, alcohol, and marijuana use. Substantially less attention has been devoted to the use of other substances (e.g., opiates, prescription medication misuse) or behavioral addiction problems (e.g., gambling disorder or excessive Internet use), which limits the generalizability of our findings to the addiction field as a whole. Also, our definition for addictive behaviors was very broad and thus included problems of various levels (e.g., alcohol use satisfying the criteria for alcohol dependence vs. marijuana use with any frequency or behavioral addictions without firm diagnostic criteria). However, this methodology must have increased the sensitivity of our analyses, that is, had we considered addictive behaviors only that are severe enough to meet diagnostic criteria, the overall support for the trauma exposure-addictive behavior relationship would have been even weaker than it is according to the present findings.

### Strengths and limitations of the study

The strengths of the present review include (1) its systematic approach, which is contrary to the vast majority of review studies in this area; (2) its wide coverage of both substance-related and behavioral addiction problems as well as a broad range of interpersonal traumata exposures, providing an opportunity to bring an inclusive perspective to research on the relationship between the two constructs; and (3) the large number of electronic databases involved in the search process without imposing limits on the date of publication.

However, several important limitations of the present study should also be acknowledged. First, we restricted our investigations to the English language literature, excluding scientific contributions in other languages. Also, each record – both during the initial screening and the data extraction phase – was evaluated by one rater only thus not providing information on the reliability of these judgements. Further, we included traumatic events only that are interpersonal in nature (not including, for example natural disasters, accidents, diseases); however, in many articles posttraumatic stress disorder was used to assess trauma exposure without further information on the source of the trauma.

In addition, the observation-level coding concerning the main outcomes of the studies yielded a more nuanced picture of the associations between interpersonal trauma exposure and addictive behaviors in different subsamples (e.g., males versus females) or with the use of different indicators when compared to a study-level description allowing only one code per a pair of variables for a single study (which we also conducted and in which case, many ‘mixed results’ codes emerged). However, since the methodological characteristics of the studies were recorded at the study level, the difference between the two approaches made it impossible to investigate the effects of the methodological variables on the study outcomes (e.g., whether the quality of assessment tools affected the emergence of significant associations).

Another disadvantage of the observation-level coding approach used in this review was that the level of detail of the analyses (and thus the number of codes assigned to a single study) was mainly related to the aims of the study authors and not the quality of the evidence or the size of the sample. That is, a disproportionately large number of codes were assigned to some studies thus giving them unreasonably large weight in the overall results. This flaw can also be a possible explanation for the moderate deviations from the main trends of our results in the case of nicotine, opiates, prescription medication, and other specific substance misuse. However, the fact that the major trends in the results were similar across the different types of trauma exposure (Table 6) or that the differences across time of trauma onset followed the hypothesized patterns (i.e. more significant positive relationships emerged in the case of childhood trauma exposure) suggests that the observation-level coding approach did not distort the results profoundly.

### Suggestions for further research

Taking stock of the literature on the exposure to traumatic interpersonal experiences and subsequent involvement in addictive behaviors and detailed analysis of the longitudinal observational studies in the field also helped us to identify major gaps and areas for further development in methodology. As mentioned, we suggest investigating the effects of traumatization on the onset of addictive behaviors by longitudinal studies following populations that are intact in terms of both trauma exposure and addictive behaviors. A less ideal but still potentially informative and more realistic approach is the prospective examination of victims immediately after experiencing trauma. However, this method also has its considerable limitations such as intervention bias (the assessment and follow-up could have intervention effects changing the natural course of adjustment to traumatization) or the ethical obligation to offer effective treatment to identified victims (again reducing the possibilities of gaining useful information about spontaneous processes), just to name a couple.

Because of the relative novelty of the concept of behavioral addictions, it is understandable that research related to them is uncommon in this literature, especially with methodologies requiring more time and resources (longitudinal studies). However, we argue for the necessity of conducting focused research on whether and how behavioral addictions are associated with trauma exposure, especially in prospective investigations. Research in populations of non-Western countries is also highly desirable.

A large proportion of null results were found in the present review, and we found some evidence to suggest that addictive behaviors may be influenced by trauma exposure within a certain time frame. It therefore seems plausible that the adverse neurobiological effects of trauma exposure [42] – as potential proximal causes of addictive behaviors – either develop only under relatively rare circumstances (e.g., very serious or prolonged traumatization) or might become less influential over time (cf., interventions based on neural plasticity [43–45]). To further elucidate this particular issue and to increase our understanding of the trauma-addiction relationship which might be largely influenced by the variability of the traumatic experience, we urge the research community to plan and conduct examinations to explicitly study more detailed trauma characteristics (e.g., intensity, duration, single versus repeated exposure, specific traumatic events) that potentially increase the vulnerability to addictive behaviors. As part of these efforts, we also recommend a more specific consideration of the exact amount of time between psychological trauma exposure, its assessment, and its (mental) health consequences. Making a clear distinction between the exposure to potentially traumatic events and actual psychological traumatization might also contribute to the elimination of the ambiguity present in the current literature concerning the causal role of trauma in the development/course of addictive behaviors.

The extent of null and significant negative associations between traumatization and addictive behaviors found in the present study also calls attention to the role of variables that may moderate this relationship. What are the specific factors that help sufferers of adverse experiences cope with trauma and avoid self-destructive behaviors? Are these factors the same or different than those that help victims to quit already initiated addictive behaviors? Among these, research might focus on the protective factors modifiable by the victim or society (e.g., maintaining former/finding new life goals, formal and informal social support from friends and family or professional organizations, resilience, nutrition) instead of variables that are much more difficult or impossible to change (e.g., race, age, immigration status, or other socioeconomic variables).

In addition, formal and explicit investigation of theoretically important mediators of the trauma-addiction association (not merely including them in the statistical models as covariates) could also be a fruitful direction for further longitudinal research, especially with the inclusion of (neuro)biological variables (operationalizing for example hypothalamic-pituitary-adrenal axis functions, noradrenergic transmission in the locus coeruleus, dopaminergic transmission in the mesocorticolimbic system [12]). Only two longitudinal studies were found in the literature that tested biological mediators between interpersonal trauma exposure and addictive behaviors [46, 47], although advanced research in this area might also help the development of biomedical tools to aid trauma victims avoid or recover from addictive disorders. We also argue for the necessity of a clearer theoretical and practical distinction between confounder and explanatory variables when controlling for covariates in multivariate analyses (cf. [48]).

Finally, the authors of the present report suggest to researchers conducting longitudinal studies to provide a clearer description of which of their variables were used at which data waves. When evaluating the studies included in the present systematic review, the coders had a surprisingly difficult time extracting this information – sometimes making it extremely time-consuming or even impossible to figure out with certainty which analyses were based on cross-sectional and which of them on longitudinal data.

## Conclusions

We can conclude that our review provides weak support for the idea that exposure to interpersonal traumata leads to the development of subsequent addictive behaviors. However, methodological choices made by researchers may account for the large number of null associations identified in relevant longitudinal research. In addition, greater precision is required to assess substantive and temporal aspects of trauma exposure. In general, the field needs to evolve beyond bivariate thinking, to test theoretically plausible mechanisms (mediators) underlying this association, as well as subpopulations (moderators) for which the association would be expected to be stronger or weaker. Conducting further investigations with the control of potential third variables (e.g., disadvantaged socioeconomic and physical environment, conflictual intimate relationships, or impaired brain function [49]) that could explain the relationship between trauma exposure and addictive behaviors without postulating a true causal link between them might also help clarify the role of trauma exposure in the development of addictive behaviors. Improving the evidence base in these ways could contribute to the development and improvement of interventions aimed to help vulnerable individuals cope with traumatic experiences and prevent the formation of addictive and other mental disorders.

## Additional files

Additional file 1: “Database search strategies”. (DOCX 23 kb)

Additional file 2: “List of articles selected for detailed coding”. (DOCX 38 kb)

Additional file 3: “Results of the detailed coding (SPSS data file)”. (SAV 127 kb)
