# Supplementary material for: Relationship between interpersonal trauma exposure and addictive behaviors: a systematic review
Source: BMC Psychiatry. 2017 May 4;17:164. doi: 10.1186/s12888-017-1323-1 (PMC5418764; doi:10.1186/s12888-017-1323-1)
Supplement: Supplementary file 1 — “Database search strategies”. (DOCX 23 kb) [file 12888_2017_1323_MOESM1_ESM.docx]

**DATABASE SEARCH STRATEGIES**

**Ovid MEDLINE(R) In-Process & Other Non-Indexed Citations and Ovid MEDLINE(R) 1946 to Present**

1. *substance-related disorders/ or exp *drinking behavior/ or exp *alcohol-related disorders/ or *amphetamine-related disorders/ or *cocaine-related disorders/ or *drug overdose/ or *inhalant abuse/ or *marijuana abuse/ or exp *opioid-related disorders/ or *phencyclidine abuse/ or *substance abuse, intravenous/ or *"tobacco use disorder"/ or exp *Smoking/

2. exp *compulsive behavior/ or exp *obsessive compulsive disorder/ or exp *obsessive behavior/ or exp *Gambling/

3. ((drug or substance or alcohol*) adj3 (dependen* or abus* or overuse or misus* or overconsumption or over consumption)).ti.

4. (addict* or abuse or binge or binging or compulsi* or obsessi* or overeat* or over eat* or gambl* or workaholi*).ti.

5. (smoking or tobacco or nicotine or cigarette* or cigar*1).ti.

6. (marijuana or marihuana or cocaine or opiate* or lsd or hash* or methamphetamine or crystal meth or cannabis or heroin or crack or ecstasy or hallucinogen* or inhalant* or psychodelics or solvent* or ((gas or glue) adj2 (sniff* or inhal*))).ti.

7. (hoarding or hoarder* or drug seeking).ti.

8. or/1-7

9. (trauma* or ptsd).ti. or exp Stress Disorders, Post-Traumatic/

10. life change events/ and stress, psychological/

11. crime/ or exp crime victims/ or exp homicide/ or exp sex offenses/ or theft/ or exp violence/ or exp war crimes/

12. exp War/

13. exp Battered Child Syndrome/

14. ((adverse adj3 experience*) or violen* or assault* or aggression or forced sex or rape or incest or molest* or neglect* or maltreat* or mistreat* or victim*).ti.

15. ((verbal or physical or psychological or spous* or elder* or partner* or emotional or sexual or domestic or wife or wives or husband* or child) adj3 abus*).ti.

16. exp Bullying/

17. (bullying or bullied or cyberbullying or cyberbullied).ti.

18. or/9-17

19. (associat* or correlat* or role or risk or concomitan* or influence* or co-morbid* or relation* or expos* or predict* or mediat*).ti,ab.

20. (study or trial or incidence or prevalence or cohort or cross section* or retrospective or prospective or follow-up or intervention* or meta analys* or systematic review or scoping review or integrative review or panel or questionnaire* or survey*).mp. or research*.pt.

21. 18 and 19 and 20

22. limit 18 to (meta analysis or observational study or systematic reviews)

23. 21 or 22

24. 8 and 23

25. limit 24 to english

**Ovid EMBASE, 1974-Current**

1. exp *addiction/ or *drinking behavior/ or exp *drug abuse/ or *drug overdose/ or *substance abuse/ or *"smoking and smoking related phenomena"/ or *bidi smoking/ or *smoking/ or *adolescent smoking/ or *smoking habit/

2. exp *obsessive compulsive disorder/ or exp *gambling/

3. (drug* or substance or alcohol*).ti.

4. (addict* or abuse or binge or binging or compulsi* or obsessi* or overeat* or over eat* or gambl* or workaholi*).ti.

5. (smoking or tobacco or nicotine or cigarette* or cigar*1).ti.

6. (marijuana or marihuana or cocaine or opiate* or lsd or hash* or methamphetamine or crystal meth or cannabis or heroin or crack or ecstasy or hallucinogen* or inhalant* or psychodelics or solvent* or ((gas or glue) adj2 (sniff* or inhal*))).ti.

7. (hoarding or hoarder* or drug seeking).ti.

8. or/1-7

9. (trauma* or ptsd).ti. or exp posttraumatic stress disorder/

10. life event/ and mental stress/

11. crime/ or crime victim/ or homicide/ or infanticide/ or sexual crime/ or theft/ or exp violence/ or war crime/ or genocide/ or holocaust/ or kidnapping/

12. war/

13. battered child syndrome/

14. ((adverse adj3 experience*) or violen* or assault* or aggression or forced sex or rape or incest or molest* or neglect* or maltreat* or mistreat* or victim*).ti.

15. ((verbal or physical or psychological or spous* or elder* or partner* or emotional or sexual or domestic or wife or wives or husband* or child) adj3 abus*).ti.

16. bullying/

17. (bullying or bullied or cyberbullying or cyberbullied).ti.

18. psychotrauma/

19. or/9-18

20. 8 and 19

21. (associat* or correlat* or role or risk or concomitan* or influence* or co-morbid* or relation* or expos* or predict* or mediat*).ti,ab.

22. (study or trial or incidence or prevalence or cohort or cross section* or retrospective or prospective or follow-up or intervention* or meta analys* or systematic review or scoping review or integrative review or panel or questionnaire* or survey*).mp.

23. 20 and 21 and 22

**Ovid PsycInfo, 1806-Current**

1. exp drug usage/

2. drinking behavior/ or exp alcohol drinking patterns/

3. exp animal drinking behavior/

4. exp addiction/ or exp gambling/

5. drug overdoses/

6. compulsions/ or exp hoarding behavior/ or obsessions/ or obsessive compulsive disorder/ or obsessive compulsive personality disorder/ or perfectionism/

7. ((drug or substance or alcohol*) adj3 (dependen* or abus* or overuse or misus* or overconsumption or over consumption)).ti,id.

8. (abus* or addict* or binge or binging or compulsi* or obsessi* or overeat* or over eat* or gambl* or workaholi*).ti,id.

9. (smoking or tobacco or nicotine or cigarette* or cigar*1).ti,id.

10. (marijuana or marihuana or cocaine or opiate* or lsd or hash* or methamphetamine or crystal meth or cannabis or heroin or crack or ecstasy or hallucinogen* or inhalant* or psychodelics or solvent* or ((gas or glue) adj2 (sniff* or inhal*))).ti,id.

11. (hoarding or hoarder* or drug seeking).ti,id.

12. or/1-11

13. (trauma* or ptsd).ti,id.

14. crime victims/ or victimization/

15. homicide/

16. sex offenses/ or exp sexual abuse/ or incest/ or pedophilia/ or sexual harassment/

17. exp violence/ or exposure to violence/ or hate crimes/ or exp partner abuse/ or exp terrorism/ or torture/ or exp war/

18. exp Posttraumatic Stress Disorder/ or exp Combat Experience/

19. child abuse/ or battered child syndrome/ or abandonment/ or child neglect/ or emotional abuse/ or patient abuse/ or verbal abuse/

20. exp bullying/

21. teasing/

22. relational aggression/

23. exp Physical Abuse/

24. ((adverse adj3 experience*) or violen* or assault* or aggression or forced sex or rape or incest or molest* or neglect* or maltreat* or mistreat* or victim*).ti,id.

25. ((verbal or physical or psychological or spous* or elder* or partner* or emotional or sexual or domestic or wife or wives or husband* or child) adj3 abus*).ti,id.

26. (bullying or bullied or cyberbullying or cyberbullied).ti,id.

27. or/13-26

28. 12 and 27

29. limit 28 to ("0400 empirical study" or "0430 followup study" or "0450 longitudinal study" or "0451 prospective study" or "0453 retrospective study" or "0830systematic review" or 1200 meta analysis or 1800 quantitative study or 2200 twin study)

30. (study or trial or incidence or prevalence or cohort or cross section* or retrospective or prospective or follow-up or intervention* or meta analys* or systematic review or scoping review or intergrative review or panel).ti,ab.

31. 28 and 29

32. 29 or 31

33. (associat* or correlat* or role or risk or concomitan* or influence* or co-morbid* or relation* or expos* or predict* or mediat*).ti,ab.

34. 32 and 33

35. limit 34 to english language

**EBSCO CINAHL Plus with Full-text, 1937-Current**

S14 S11 AND S12 AND S13

S13 ( (study or trial or incidence or prevalence or cohort or cross section* or retrospective or prospective or follow-up or intervention* or meta analys* or systematic review or scoping review or intergrative review or panel or questionnaire* or survey*) ) OR PT research*

S12 ( TI (associat* or correlat* or role or risk or concomitan* or influence* or co-morbid* or relation* or expos* or predict* or mediat*) ) OR ( AB (associat* or correlat* or role or risk or concomitan* or influence* or co-morbid* or relation* or expos* or predict* or mediat*) )

S11 S4 AND S10

S10 S5 OR S6 OR S7 OR S8 OR S9

S9 TI (bullying or bullied or cyberbullying or cyberbullied)

S8 TI ((verbal or physical or psychological or spous* or elder* or partner* or emotional or sexual or domestic or wife or wives or husband* or child) N3 abus*)

S7 TI ((adverse N3 experience*) or violen* or assault* or aggression or forced sex or rape or incent or molest* or neglect* or maltreat* or mistreat* or victim*) S6 (MH "Life Change Events+") AND (MH "Stress, Psychological+") OR (MH "Crime+") OR (MH "Crime Victims") OR (MH "Patient Assault") OR (MH "Verbal Abuse") OR (MH "Torture") OR (MH "Bullying")

S5 TI (trauma* or ptsd) OR (MH "Stress Disorders, Post-Traumatic+") OR (MH "War+")

S4 S1 OR S2 OR S3

S3 TI (solvent* or ((gas or glue) N2 (sniff* or inhal*)))

S2 TI (drug* or substance or alcohol* or addict* or binge or binging or compulsi* or obsessi* or overeat* or over eat* or gambl* or workaholi* or smoking or tobacco or nicotine or cigarette* or cigar* or marijuana or marihuana or cocaine or opiate* or lsd or hash* or methamphetamine or crystal meth or cannabis or heroin or crack or ecstasy or hallucinogen* or inhalant* or psychodelics or solvent* or hoarding or hoarder* or drug seeking)

S1 (MH "Compulsive Behavior") OR (MH "Obsessive-Compulsive Disorder") OR (MH "Gambling") OR (MH "Substance Use Disorders") OR (MH "Alcohol-Related Disorders") OR (MH "Substance Abuse") OR (MH "Alcohol Abuse") OR (MH "Alcoholic Intoxication") OR (MH "Alcoholism") OR (MH "Binge Drinking") OR (MH "Inhalant Abuse") OR (MH "Substance Abuse, Intravenous") OR (MH "Substance Abuse, Perinatal") OR (MH "Smoking")

**EBSCO Violence and Abuse Abstracts, 1984-Current**

S12 S6 AND S11

S11 S7 OR S8 OR S9 OR S10

S10 TI (bullying or bullied or cyberbullying or cyberbullied)

S9 ((verbal or physical or psychological or spous* or elder* or partner* or emotional or sexual or domestic or wife or wives or husband* or child) N3 abus*)

S8 ((adverse N3 experience*) or violen* or assault* or aggression or forced sex or rape or incest or molest* or neglect* or maltreat* or mistreat* or victim*)

S7 trauma*

S6 S1 OR S2 OR S3 OR S4 OR S5

S5 TI (hoarding or hoarder* or drug seeking) or SU (hoarding or hoarder* or drug seeking)

S4 TI (marijuana or marihuana or cocaine or opiate* or lsd or hash* or methamphetamine or crystal meth or cannabis or heroin or crack or ecstasy or hallucinogen* or inhalant* or psychodelics or solvent* or ((gas or glue) N2 (sniff* or inhal*))) OR SU (marijuana or marihuana or cocaine or opiate* or lsd or hash* or methamphetamine or crystal meth or cannabis or heroin or crack or ecstasy or hallucinogen* or inhalant* or psychodelics or solvent* or ((gas or glue) N2 (sniff* or inhal*)))

S3 TI (smoking or tobacco or nicotine or cigarette* or cigar*) or SU (smoking or tobacco or nicotine or cigarette* or cigar*)

S2 TI (addict* or binge or binging or compulsi* or obsessi* or overeat* or over eat* or gambl* or workaholi*) or SU (addict* or binge or binging or compulsi* or obsessi* or overeat* or over eat* or gambl* or workaholi*)

S1 TI (drug* or substance or alcohol*) or SU (drug or substance or alcohol*)

**ProQuest PILOTS (Published International Literature on Traumatic Stress), 1871-Current**

NOTE: No terms were added related to trauma because everything in this database should have some connection to traumatic stress.

ti((drug OR substance OR alcohol*) AND (dependen* OR abus* OR overuse OR misus* OR overconsumption OR over consumption)) OR ti(abus* OR addict* OR binge OR binging OR compulsi* OR obsessi* OR overeat* OR over eat* OR gambl* OR workaholi*) OR ti(smoking OR tobacco OR nicotine OR cigar*) OR ti((marijuana or marihuana or cocaine or opiate* or lsd or hash* or methamphetamine or crystal meth or cannabis or heroin or crack or ecstasy or hallucinogen* or inhalant* or psychedelic* or solvent* OR (gas or glue) and (sniff* or inhal*))) OR ti((hoarding or hoarder* or "drug seeking" OR alcohol or ethanol))

**Scopus, 1960-Current**

TITLE((drug W/3 dependen*) OR (drug W/3 abus*) OR (drug W/3 overuse) OR (drug W/3 misus*) OR (drug* W/3 overconsum*) OR (drug* W/3 consum*) OR (substance W/3 dependen*) OR (substance W/3 abus*) OR (substance W/3 overus*) OR (substance W/3 misus*) OR (substance W/3 overconsum*) OR (substance W/3 consum*) OR (alcohol* W/3 depend*) OR (alcohol* W/3 abus*) OR (alcohol W/3 overuse) OR (alcohol W/3 misus*) OR (alcohol* W/3 overconsum*) OR (alcohol* W/3 consum*) OR addict* OR binge OR binging OR compulsi* OR obsessi* OR overeat* OR {over eat*} OR gambl* OR workaholi* OR smoking OR tobacco OR nicotine OR cigarette* OR cigar* OR marijuana OR marihuana OR cocaine OR opiate* OR lsd OR hash OR hashish OR methamphetamine OR {crystal meth} OR cannabis OR heroin OR ecstasy OR hallucinogen* OR inhalant* OR psychodelics OR solvent* OR (gas W/2 sniff*) OR (glue W/3 sniff*) OR (glue W/2 inhal*) OR (gas W/2 inhal*) OR hoard* OR {drug seeking}) AND TITLE((verbal W/3 abus*) OR (physical W/3 abus*) OR (psychological W/3 abus*) OR (adverse W/3 experience*) OR violen* OR assault* OR aggression OR {forced sex} OR rape OR incest OR molest* OR neglect* OR maltreat* OR mistreat* OR victim* OR (spous* W/3 abus*) OR (wife W/3 abus*) OR (wives W/3 abus*) OR (elder* W/3 abus*) OR (partner W/3 abus*) OR (husband W/3 abus*) OR (child W/3 abus*) OR (sexual W/3 abus*) OR (domestic W/3 abus*) OR bullying OR bullied OR cyberbullying OR cyberbullied OR trauma*) AND (TITLE(associat* OR correlat* OR role OR risk OR concomitan* OR influenc* OR {co morbid} OR relation* OR expos* OR predict* OR mediat*) OR ABS(associat* OR correlat* OR role OR risk OR concomitan* OR influenc* OR {co morbid} OR relation* OR expos* OR predict* OR mediat*) OR TITLE-ABS-KEY(study OR trial OR incidence OR prevalence OR cohort OR {cross section*} OR retrospective OR prospective OR {follow up} OR intervention* OR {meta analys*} OR {systematic review} OR {scoping review} OR {integrative review} OR panel OR questionnaire* OR survey*))
