# Supplementary material for: Relationship between interpersonal trauma exposure and addictive behaviors: a systematic review
Source: BMC Psychiatry. 2017 May 4;17:164. doi: 10.1186/s12888-017-1323-1 (PMC5418764; doi:10.1186/s12888-017-1323-1)
Supplement: Supplementary file 2 — “List of articles selected for detailed coding”. (DOCX 38 kb) [file 12888_2017_1323_MOESM2_ESM.docx]

**LIST OF ARTICLES SELECTED FOR DETAILED CODING**

1. Ackard, D. M., Eisenberg, M. E., & Neumark-Sztainer, D. (2007). Long-term impact of adolescent dating violence on the behavioral and psychological health of male and female youth. *Journal of Pediatrics, 151*(5), 476-481.
2. Alegria, M., Vera, M., Shrout, P., Canino, G., Lai, S., Albizu, C., . . . Rusch, D. (2004). Understanding hard-core drug use among urban Puerto Rican women in high-risk neighborhoods. *Addictive Behaviors, 29*(4), 643-664.
3. Bachrach, R. L., & Read, J. P. (2012). The Role of Posttraumatic Stress and Problem Alcohol Involvement in University Academic Performance. *Journal of Clinical Psychology, 68*(7), 843-859.
4. Bailey, J. A., & McCloskey, L. A. (2005). Pathways to adolescent substance use among sexually abused girls. *Journal of Abnormal Child Psychology, 33*(1), 39-53.
5. Beckham, J. C., Calhoun, P. S., Dennis, M. F., Wilson, S. M., & Dedert, E. A. (2013). Predictors of lapse in first week of smoking abstinence in PTSD and non-PTSD smokers. *Nicotine & tobacco research, 15*(6), 1122-1129.
6. Beckham, J. C., Feldman, M. E., Vrana, S. R., Mozley, S. L., Erkanli, A., Clancy, C. P., & Rose, J. E. (2005). Immediate Antecedents of Cigarette Smoking in Smokers With and Without Posttraumatic Stress Disorder: A Preliminary Study. *Experimental and Clinical Psychopharmacology, 13*(3), 219-228.
7. Beckham, J. C., Wiley, M. T., Miller, S. C., Dennis, M. F., Wilson, S. M., McClernon, F. J., & Calhoun, P. S. (2008). Ad lib smoking in post-traumatic stress disorder: an electronic diary study. *Nicotine & tobacco research, 10*(7), 1149-1157.
8. Bonn-Miller, M. O., Vujanovic, A. A., & Drescher, K. D. (2011). Cannabis use among military veterans after residential treatment for posttraumatic stress disorder. *Psychology of Addictive Behaviors, 25*(3), 485-491.
9. Boscarino, J. A., Adams, R. E., & Galea, S. (2006). Alcohol use in New York after the terrorist attacks: a study of the effects of psychological trauma on drinking behavior. *Addictive Behaviors, 31*(4), 606-621.
10. Boscarino, J. A., Kirchner, H. L., Hoffman, S. N., Sartorius, J., & Adams, R. E. (2011). PTSD and alcohol use after the World Trade Center attacks: a longitudinal study. *Journal of Traumatic Stress, 24*(5), 515-525.
11. Brady, S. S., Tschann, J. M., Pasch, L. A., Flores, E., & Ozer, E. J. (2008). Violence involvement, substance use, and sexual activity among Mexican-American and European-American adolescents. *Journal of Adolescent Health, 43*(3), 285-295.
12. Breslau, N., Davis, G. C., & Schultz, L. R. (2003). Posttraumatic stress disorder and the incidence of nicotine, alcohol, and other drug disorders in persons who have experienced trauma. *Archives of General Psychiatry, 60*(3), 289-294.
13. Brown, A., Cosgrave, E., Killackey, E., Purcell, R., Buckby, J., & Yung, A. R. (2009). The longitudinal association of adolescent dating violence with psychiatric disorders and functioning. *Journal of Interpersonal Violence, 24*(12), 1964-1979.
14. Brown, P. J. (2000). Outcome in female patients with both substance use and Post-Traumatic Stress Disorders. *Alcoholism Treatment Quarterly, 18*(3), 127-135.
15. Brown, P. J., Stout, R. L., & Mueller, T. (1996). Posttraumatic stress disorder and substance abuse relapse among women: a pilot study. *Psychology of Addictive Behaviors, 10*(2), 124-128.
16. Burgdorf, K., Chen, X., Walker, T., Porowski, A., & Herrell, J. M. (2004). The Prevalence and Prognostic Significance of Sexual Abuse in Substance Abuse Treatment of Women. *Addictive Disorders and their Treatment, 3*(1), 1-13.
17. Burns, M. N., Lehman, K. A., Milby, J. B., Wallace, D., & Schumacher, J. E. (2010). Do PTSD symptoms and course predict continued substance use for homeless individuals in contingency management for cocaine dependence? *Behaviour Research and Therapy, 48*(7), 588-598.
18. Cachelin, F. M., Striegel-Moore, R. H., Elder, K. A., Pike, K. M., Wilfley, D. E., & Fairburn, C. G. (1999). Natural course of a community sample of women with binge eating disorder. *International Journal of Eating Disorders, 25*(1), 45-54.
19. Castro, F. G., Brook, J. S., Brook, D. W., & Rubenstone, E. (2006). Paternal, perceived maternal, and youth risk factors as predictors of youth stage of substance use a longitudinal study. *Journal of Addictive Diseases, 25*(2), 65-75.
20. Cerda, M., Tracy, M., & Galea, S. (2011). A prospective population based study of changes in alcohol use and binge drinking after a mass traumatic event. *Drug and Alcohol Dependence, 115*(1-2), 1-8.
21. Cerda, M., Vlahov, D., Tracy, M., & Galea, S. (2008). Alcohol use trajectories among adults in an urban area after a disaster: evidence from a population-based cohort study. *Addiction, 103*(8), 1296-1307.
22. Charney, D. A., Palacios-Boix, J., & Gill, K. J. (2007). Sexual abuse and the outcome of addiction treatment. *American Journal on Addictions, 16*(2), 93-100.
23. Chauhan, P., & Widom, C. S. (2012). Childhood maltreatment and illicit drug use in middle adulthood: the role of neighborhood characteristics. *Development and Psychopathology, 24*(3), 723-738.
24. Chilcoat, H. D., & Breslau, N. (1998). Posttraumatic stress disorder and drug disorders: Testing causal pathways. *Archives of General Psychiatry, 55*(10), 913-917.
25. Chiodo, D., Wolfe, D. A., Crooks, C., Hughes, R., & Jaffe, P. (2009). Impact of sexual harassment victimization by peers on subsequent adolescent victimization and adjustment: a longitudinal study. *Journal of Adolescent Health, 45*(3), 246-252.
26. Choi, K. W., Abler, L. A., Watt, M. H., Eaton, L. A., Kalichman, S. C., Skinner, D., . . . Sikkema, K. J. (2014). Drinking before and after pregnancy recognition among South African women: the moderating role of traumatic experiences. *BMC Pregnancy and Childbirth, 14, 97*.
27. Chu, D. C. (2012). The links between religiosity, childhood sexual abuse, and subsequent marijuana use: an empirical inquiry of a sample of female college students. *International Journal of Offender Therapy and Comparative Criminology, 56*(6), 937-954.
28. Chung, E. K., Nurmohamed, L., Mathew, L., Elo, I. T., Coyne, J. C., & Culhane, J. F. (2010). Risky health behaviors among mothers-to-be: the impact of adverse childhood experiences. *Academic Pediatrics, 10*(4), 245-251.
29. Cisler, J. M., Amstadter, A. B., Begle, A. M., Resnick, H. S., Danielson, C. K., Saunders, B. E., & Kilpatrick, D. G. (2011). A prospective examination of the relationships between PTSD, exposure to assaultive violence, and cigarette smoking among a national sample of adolescents. *Addictive Behaviors, 36*(10), 994-1000.
30. Cisler, J. M., Amstadter, A. B., Begle, A. M., Resnick, H. S., Danielson, C. K., Saunders, B. E., & Kilpatrick, D. G. (2011). PTSD symptoms, potentially traumatic event exposure, and binge drinking: a prospective study with a national sample of adolescents. *Journal of Anxiety Disorders, 25*(7), 978-987.
31. Cisler, J. M., Begle, A. M., Amstadter, A. B., Resnick, H. S., Danielson, C. K., Saunders, B. E., & Kilpatrick, D. G. (2012). Exposure to interpersonal violence and risk for PTSD, depression, delinquency, and binge drinking among adolescents: data from the NSA-R. *Journal of Traumatic Stress, 25*(1), 33-40.
32. Clark, D. B., De Bellis, M. D., Lynch, K. G., Cornelius, J. R., & Martin, C. S. (2003). Physical and sexual abuse, depression and alcohol use disorders in adolescents: onsets and outcomes. *Drug and Alcohol Dependence, 69*(1), 51-60.
33. Cohn, A., Hagman, B. T., Moore, K., Mitchell, J., & Ehlke, S. (2014). Does negative affect mediate the relationship between daily PTSD symptoms and daily alcohol involvement in female rape victims? Evidence from 14 days of interactive voice response assessment. *Psychology of Addictive Behaviors, 28*(1), 114-126.
34. Cornelius, J. R., Kirisci, L., Reynolds, M., Clark, D. B., Hayes, J., & Tarter, R. (2010). PTSD contributes to teen and young adult cannabis use disorders. *Addictive Behaviors, 35*(2), 91-94.
35. Danielson, C. K., Amstadter, A. B., Dangelmaier, R. E., Resnick, H. S., Saunders, B. E., & Kilpatrick, D. G. (2009). Trauma-related risk factors for substance abuse among male versus female young adults. *Addictive Behaviors, 34*(4), 395-399.
36. DeBeck, K., Kerr, T., Marshall, B. D., Simo, A., Montaner, J., & Wood, E. (2013). Risk factors for progression to regular injection drug use among street-involved youth in a Canadian setting. *Drug and Alcohol Dependence, 133*(2), 468-472.
37. Dembo, R., Wareham, J., & Schmeidler, J. (2007). A longitudinal study of cocaine use among juvenile arrestees. *Journal of Child & Adolescent Substance Abuse, 17*(1), 83-109.
38. Dembo, R., Williams, L., Fagan, J., & Schmeidler, J. (1993). The relationships of substance abuse and other delinquency over time in a sample of juvenile detainees. *Criminal Behaviour and Mental Health, 3*(3), 158-179.
39. Doherty, E. E., Robertson, J. A., Green, K. M., Fothergill, K. E., & Ensminger, M. E. (2012). A longitudinal study of substance use and violent victimization in adulthood among a cohort of urban African Americans. *Addiction, 107*(2), 339-348.
40. Earnshaw, V. A., Rosenthal, L., Carroll-Scott, A., Peters, S. M., McCaslin, C., & Ickovics, J. R. (2014). Teacher involvement as a protective factor from the association between race-based bullying and smoking initiation. *Social Psychology of Education*, 1-13.
41. El-Bassel, N., Gilbert, L., Wu, E., Go, H., & Hill, J. (2005). Relationship between drug abuse and intimate partner violence: a longitudinal study among women receiving methadone. *American Journal of Public Health, 95*(3), 465-470.
42. Elliott, J. C., Stohl, M., Wall, M. M., Keyes, K. M., Goodwin, R. D., Skodol, A. E., . . . Hasin, D. S. (2014). The risk for persistent adult alcohol and nicotine dependence: the role of childhood maltreatment. *Addiction, 109*(5), 842-850.
43. Elwyn, L. J. (2011). *Adult memory and appraisal of maltreatment in childhood: Implications for problem substance use.* (Dissertation), State University of New York, New York.
44. Epstein, J. N., Saunders, B. E., Kilpatrick, D. G., & Resnick, H. S. (1998). PTSD as a mediator between childhood rape and alcohol use in adult women. *Child Abuse and Neglect, 22*(3), 223-234.
45. Eriksen, W. (2006). Work factors as predictors of smoking relapse in nurses' aides. *International Archives of Occupational and Environmental Health, 79*(3), 244-250.
46. Exner-Cortens, D., Eckenrode, J., & Rothman, E. (2013). Longitudinal Associations Between Teen Dating Violence Victimization and Adverse Health Outcomes. *Pediatrics, 131*(1), 71-78.
47. Fergusson, D. M., Boden, J. M., & Horwood, L. J. (2008). The developmental antecedents of illicit drug use: evidence from a 25-year longitudinal study. *Drug and Alcohol Dependence, 96*(1-2), 165-177.
48. Fiorentine, R., Pilati, M. L., & Hillhouse, M. P. (1999). Drug treatment outcomes: investigating the long-term effects of sexual and physical abuse histories. *Journal of Psychoactive Drugs, 31*(4), 363-372.
49. Fishbein, D., Novak, S. P., Krebs, C., Warner, T., & Hammond, J. (2011). The mediating effect of depressive symptoms on the relationship between traumatic childhood experiences and drug use initiation. *Addictive Behaviors, 36*(5), 527-531.
50. Ford, J. D., Hawke, J., Alessi, S., Ledgerwood, D., & Petry, N. (2007). Psychological trauma and PTSD symptoms as predictors of substance dependence treatment outcomes. *Behaviour Research and Therapy, 45*(10), 2417-2431.
51. Forman-Hoffman, V., Riley, W., & Pici, M. (2005). Acute impact of the September 11 tragedy on smoking and early relapse rates among smokers attempting to quit. *Psychology of Addictive Behaviors, 19*(3), 277-283.
52. Foshee, V. A., Reyes, H. L., Gottfredson, N. C., Chang, L. Y., & Ennett, S. T. (2013). A longitudinal examination of psychological, behavioral, academic, and relationship consequences of dating abuse victimization among a primarily rural sample of adolescents. *Journal of Adolescent Health, 53*(6), 723-729.
53. Frank, D. A., Rose-Jacobs, R., Crooks, D., Cabral, H. J., Gerteis, J., Hacker, K. A., . . . Heeren, T. (2011). Adolescent initiation of licit and illicit substance use: Impact of intrauterine exposures and post-natal exposure to violence. *Neurotoxicology and Teratology, 33*(1), 100-109.
54. Galaif, E. R., Stein, J. A., Newcomb, M. D., & Bernstein, D. P. (2001). Gender differences in the prediction of problem alcohol use in adulthood: exploring the influence of family factors and childhood maltreatment. *Journal of Studies on Alcohol, 62*(4), 486-493. Retrieved from http://www.jsad.com/jsad/downloadarticle/Gender_Differences_in_the_Prediction_of_Problem_Alcohol_Use_in_Adulthood_E/1487.pdf
55. Gamez-Guadix, M., Orue, I., Smith, P. K., & Calvete, E. (2013). Longitudinal and reciprocal relations of cyberbullying with depression, substance use, and problematic internet use among adolescents. *Journal of Adolescent Health, 53*(4), 446-452.
56. Gao, W., Paterson, J., Abbott, M., Carter, S., Iusitini, L., & McDonald-Sundborn, G. (2010). Impact of current and past intimate partner violence on maternal mental health and behaviour at 2 years after childbirth: evidence from the Pacific Islands Families Study. *Australian and New Zealand Journal of Psychiatry, 44*(2), 174-182.
57. Gavin, A. R., Hill, K. G., Hawkins, J. D., & Maas, C. (2011). The role of maternal early-life and later-life risk factors on offspring low birth weight: findings from a three-generational study. *Journal of Adolescent Health, 49*(2), 166-171.
58. Gidycz, C. A., Loh, C., Lobo, T., Rich, C., Lynn, S. J., & Pashdag, J. (2007). Reciprocal relationships among alcohol use, risk perception, and sexual victimization: a prospective analysis. *Journal of American College Health, 56*(1), 5-14.
59. Gilbert, L. (2009). *Substance use and intimate partner violence among low income, urban women seeking care in an emergency department.* (Dissertation), Columbia University, New York
60. Glasner-Edwards, S., Mooney, L. J., Ang, A., Hillhouse, M., & Rawson, R. (2013). Does posttraumatic stress disorder affect post-treatment methamphetamine use? *Journal of Dual Diagnosis, 9*(2), 123-128.
61. Greenfield, S. F., Kolodziej, M. E., Sugarman, D. E., Muenz, L. R., Vagge, L. M., He, D. Y., & Weiss, R. D. (2002). History of abuse and drinking outcomes following inpatient alcohol treatment: a prospective study. *Drug and Alcohol Dependence, 67*(3), 227-234.
62. Haller, M. (2014). *Disentangling the directions of influence among trauma exposure, posttraumatic stress disorder symptoms, and alcohol and drug problems.* (Dissertation), Arizona State University, Phoenix, AZ.
63. Hedtke, K. A., Ruggiero, K. J., Fitzgerald, M. M., Zinzow, H. M., Saunders, B. E., Resnick, H. S., & Kilpatrick, D. G. (2008). A longitudinal investigation of interpersonal violence in relation to mental health and substance use. *Journal of Consulting and Clinical Psychology, 76*(4), 633-647.
64. Helzer, J. E. (1984). The impact of combat on later alcohol use by Vietnam veterans. *Journal of Psychoactive Drugs, 16*(2), 183-191.
65. Hermes, E. D., Wells, T. S., Smith, B., Boyko, E. J., Gackstetter, G. G., Miller, S. C., . . . Millennium Cohort Study, T. (2012). Smokeless tobacco use related to military deployment, cigarettes and mental health symptoms in a large, prospective cohort study among US service members. *Addiction, 107*(5), 983-994.
66. Herrenkohl, T. I., Hong, S., Klika, J., Herrenkohl, R. C., & Russo, M. (2013). Developmental impacts of child abuse and neglect related to adult mental health, substance use, and physical health. *Journal of Family Violence, 28*(2), 191-199.
67. Hien, D. A., Nunes, E., Levin, F. R., & Fraser, D. (2000). Posttraumatic stress disorder and short-term outcome in early methadone treatment. *Journal of Substance Abuse Treatment, 19*(1), 31-37.
68. Hooper, R., Rona, R. J., Jones, M., Fear, N. T., Hull, L., & Wessely, S. (2008). Cigarette and alcohol use in the UK Armed Forces, and their association with combat exposures: A prospective study. *Addictive Behaviors, 33*(8), 1067-1071.
69. Hyman, S. M., Paliwal, P., Chaplin, T. M., Mazure, C. M., Rounsaville, B. J., & Sinha, R. (2008). Severity of childhood trauma is predictive of cocaine relapse outcomes in women but not men. *Drug and Alcohol Dependence, 92*(1-3), 208-216.
70. James, L. M., Van Kampen, E., Miller, R. D., & Engdahl, B. E. (2013). Risk and protective factors associated with symptoms of post-traumatic stress, depression, and alcohol misuse in OEF/OIF veterans. *Military Medicine, 178*(2), 159-165.
71. Jasinski, J. L., Williams, L. M., & Siegel, J. (2000). Childhood physical and sexual abuse as risk factors for heavy drinking among African-American women: a prospective study. *Child Abuse and Neglect, 24*(8), 1061-1071.
72. Jones, D. J., Lewis, T., Litrownik, A., Thompson, R., Proctor, L. J., Isbell, P., . . . Runyan, D. (2013). Linking childhood sexual abuse and early adolescent risk behavior: the intervening role of internalizing and externalizing problems. *Journal of Abnormal Child Psychology, 41*(1), 139-150.
73. Jones, D. J., Runyan, D. K., Lewis, T., Litrownik, A. J., Black, M. M., Wiley, T., . . . Nagin, D. S. (2010). Trajectories of childhood sexual abuse and early adolescent HIV/AIDS risk behaviors: the role of other maltreatment, witnessed violence, and child gender. *Journal of Clinical Child and Adolescent Psychology, 39*(5), 667-680.
74. Joseph, N. P., Augustyn, M., Cabral, H., & Frank, D. A. (2006). Preadolescents' report of exposure to violence: association with friends' and own substance use. *Journal of Adolescent Health, 38*(6), 669-674.
75. Jun, H. J., Austin, S. B., Wylie, S. A., Corliss, H. L., Jackson, B., Spiegelman, D., . . . Wright, R. J. (2010). The mediating effect of childhood abuse in sexual orientation disparities in tobacco and alcohol use during adolescence: results from the Nurses' Health Study II. *Cancer Causes and Control, 21*(11), 1817-1828.
76. Kang, S.-Y., Magura, S., Laudet, A., & Whitney, S. (1999). Adverse effect of child abuse victimization among substance-using women in treatment. *Journal of Interpersonal Violence, 14*(6), 657-670.
77. Kaufman, J., Yang, B. Z., Douglas-Palumberi, H., Crouse-Artus, M., Lipschitz, D., Krystal, J. H., & Gelernter, J. (2007). Genetic and environmental predictors of early alcohol use. *Biological Psychiatry, 61*(11), 1228-1234.
78. Kaysen, D., Atkins, D. C., Simpson, T. L., Stappenbeck, C. A., Blayney, J. A., Lee, C. M., & Larimer, M. E. (2014). Proximal relationships between PTSD symptoms and drinking among female college students: Results from a daily monitoring study. *Psychology of Addictive Behaviors, 28*(1), 62-73.
79. Kaysen, D., Neighbors, C., Martell, J., Fossos, N., & Larimer, M. E. (2006). Incapacitated rape and alcohol use: a prospective analysis. *Addictive Behaviors, 31*(10), 1820-1832.
80. Kehle, S. M., Ferrier-Auerbach, A. G., Meis, L. A., Arbisi, P. A., Erbes, C. R., & Polusny, M. A. (2012). Predictors of postdeployment alcohol use disorders in National Guard soldiers deployed to Operation Iraqi Freedom. *Psychology of Addictive Behaviors, 26*(1), 42-50.
81. Kilpatrick, D. G., Acierno, R., Resnick, H. S., Saunders, B. E., & Best, C. L. (1997). A 2-year longitudinal analysis of the relationships between violent assault and substance use in women. *Journal of Consulting and Clinical Psychology, 65*(5), 834-847.
82. Kirisci, L., Dunn, M. G., Mezzich, A. C., & Tarter, R. E. (2001). Impact of parental substance use disorder and child neglect severity on substance use involvement in male offspring. *Prevention Science, 2*(4), 241-255.
83. Kliewer, W., & Zaharakis, N. (2013). Community violence exposure, coping, and problematic alcohol and drug use among urban, female caregivers: A prospective study. *Personality and Individual Differences, 55*(4), 361-366.
84. Kline, A., Weiner, M. D., Ciccone, D. S., Interian, A., Hill, L. S., & Losonczy, M. (2014). Increased risk of alcohol dependency in a cohort of national guard troops with PTSD: A longitudinal study. *Journal of Psychiatric Research, 50*(1), 18-25.
85. La Flair, L. N., Reboussin, B. A., Storr, C. L., Letourneau, E., Green, K. M., Mojtabai, R., . . . Crum, R. M. (2013). Childhood abuse and neglect and transitions in stages of alcohol involvement among women: a latent transition analysis approach. *Drug and Alcohol Dependence, 132*(3), 491-498.
86. Lansford, J. E., Dodge, K. A., Pettit, G. S., & Bates, J. E. (2010). Does physical abuse in early childhood predict substance use in adolescence and early adulthood? *Child Maltreatment, 15*(2), 190-194.
87. Lewis, T. L., Kotch, J., Wiley, T. R., Litrownik, A. J., English, D. J., Thompson, R., . . . Dubowitz, H. (2011). Internalizing problems: a potential pathway from childhood maltreatment to adolescent smoking. *Journal of Adolescent Health, 48*(3), 247-252.
88. Liebschutz, J. M., Geier, J. L., Horton, N. J., Chuang, C. H., & Samet, J. H. (2005). Physical and sexual violence and health care utilization in HIV-infected persons with alcohol problems. *AIDS Care, 17*(5), 566-578.
89. Lindgren, K. P., Neighbors, C., Blayney, J. A., Mullins, P. M., & Kaysen, D. (2012). Do drinking motives mediate the association between sexual assault and problem drinking? *Addictive Behaviors, 37*(3), 323-326.
90. Lo, C. C., & Cheng, T. C. (2007). The impact of childhood maltreatment on young adults' substance abuse. *American Journal of Drug and Alcohol Abuse, 33*(1), 139-146.
91. Maguen, S., Litz, B. T., Wang, J. L., & Cook, M. (2004). The Stressors and Demands of Peacekeeping in Kosovo: Predictors of Mental Health Response. *Military Medicine, 169*(3), 198-206.
92. Maguen, S., Stalnaker, M., McCaslin, S., & Litz, B. T. (2009). PTSD subclusters and functional impairment in Kosovo peacekeepers. *Military Medicine, 174*(8), 779-785.
93. Martino, S. C., Collins, R. L., & Ellickson, P. L. (2005). Cross-lagged relationships between substance use and intimate partner violence among a sample of young adult women. *Journal of Studies on Alcohol, 66*(1), 139-148.
94. McCart, M. R., Zajac, K., Kofler, M. J., Smith, D. W., Saunders, B. E., & Kilpatrick, D. G. (2012). Longitudinal examination of PTSD symptoms and problematic alcohol use as risk factors for adolescent victimization. *Journal of Clinical Child and Adolescent Psychology, 41*(6), 822-836.
95. Messina, N., Marinelli-Casey, P., Hillhouse, M., Rawson, R., Hunter, J., & Ang, A. (2008). Childhood adverse events and methamphetamine use among men and women. *Journal of Psychoactive Drugs, Suppl 5*, 399-409.
96. Messman-Moore, T. L., Ward, R. M., & Brown, A. L. (2009). Substance use and PTSD symptoms impact the likelihood of rape and revictimization in college women. *Journal of Interpersonal Violence, 24*(3), 499-521.
97. Mills, R., Alati, R., Strathearn, L., & Najman, J. M. (2014). Alcohol and tobacco use among maltreated and non-maltreated adolescents in a birth cohort. *Addiction, 109*(4), 672-680.
98. Moore, S. E., Norman, R. E., Sly, P. D., Whitehouse, A. J. O., Zubrick, S. R., & Scott, J. (2014). Adolescent peer aggression and its association with mental health and substance use in an Australian cohort. *Journal of Adolescence, 37*(1), 11-21.
99. Mouilso, E. R., Fischer, S., & Calhoun, K. S. (2012). A prospective study of sexual assault and alcohol use among first-year college women. *Violence and Victims, 27*(1), 78-94.
100. Mrug, S., & Windle, M. (2009). Initiation of alcohol use in early adolescence: links with exposure to community violence across time. *Addictive Behaviors, 34*(9), 779-781.
101. Murphy, S. A., Beaton, R. D., Pike, K. C., & Johnson, L. (1999). Occupational stressors, stress responses, and alcohol consumption among professional firefighters: A prospective, longitudinal analysis. *International Journal of Stress Management, 6*(3), 179-196.
102. Najdowski, C. J., & Ullman, S. E. (2009). Prospective effects of sexual victimization on PTSD and problem drinking. *Addictive Behaviors, 34*(11), 965-968.
103. Newcomb, M. D., & Carmona, J. V. (2004). Adult trauma and HIV status among Latinas: effects upon psychological adjustment and substance use. *AIDS and Behavior, 8*(4), 417-428.
104. Niemela, S., Brunstein-Klomek, A., Sillanmaki, L., Helenius, H., Piha, J., Kumpulainen, K., . . . Sourander, A. (2011). Childhood bullying behaviors at age eight and substance use at age 18 among males. A nationwide prospective study. *Addictive Behaviors, 36*(3), 256-260.
105. Norman, S. B., Tate, S. R., Anderson, K. G., & Brown, S. A. (2007). Do trauma history and PTSD symptoms influence addiction relapse context? *Drug and Alcohol Dependence, 90*(1), 89-96.
106. North, C. S., Pfefferbaum, B., Kawasaki, A., Lee, S., & Spitznagel, E. L. (2011). Psychosocial adjustment of directly exposed survivors 7 years after the Oklahoma City bombing. *Comprehensive Psychiatry, 52*(1), 1-8.
107. Nowotny, K. M., & Graves, J. L. (2013). Substance use and intimate partner violence victimization among white, African American, and Latina women. *Journal of Interpersonal Violence, 28*(17), 3301-3318.
108. Oshri, A., Rogosch, F. A., Burnette, M. L., & Cicchetti, D. (2011). Developmental pathways to adolescent cannabis abuse and dependence: child maltreatment, emerging personality, and internalizing versus externalizing psychopathology. *Psychology of Addictive Behaviors, 25*(4), 634-644.
109. Ouimette, P. C., Ahrens, C., Moos, R. H., & Finney, J. W. (1997). Posttraumatic stress disorder in substance abuse patients: Relationship to 1-year posttreatment outcomes. *Psychology of Addictive Behaviors, 11*(1), 34-47.
110. Ouimette, P., Coolhart, D., Funderburk, J. S., Wade, M., & Brown, P. J. (2007). Precipitants of first substance use in recently abstinent substance use disorder patients with PTSD. *Addictive Behaviors, 32*(8), 1719-1727.
111. Pedersen, W., & Skrondal, A. (1996). Alcohol and sexual victimization: a longitudinal study of Norwegian girls. *Addiction, 91*(4), 565-581.
112. Peirce, J. M., Brooner, R. K., Kolodner, K., Schacht, R. L., & Kidorf, M. S. (2013). Prospective effects of traumatic event re-exposure and post-traumatic stress disorder in syringe exchange participants. *Addiction, 108*(1), 146-153.
113. Pirard, S., Sharon, E., Kang, S. K., Angarita, G. A., & Gastfriend, D. R. (2005). Prevalence of physical and sexual abuse among substance abuse patients and impact on treatment outcomes. *Drug and Alcohol Dependence, 78*(1), 57-64.
114. Price, R. K., Risk, N. K., Haden, A. H., Lewis, C. E., & Spitznagel, E. L. (2004). Post-traumatic stress disorder, drug dependence, and suicidality among male Vietnam veterans with a history of heavy drug use. *Drug and Alcohol Dependence, 76 Suppl*, S31-43.
115. Read, J. P., Brown, P. J., & Kahler, C. W. (2004). Substance use and posttraumatic stress disorders: Symptom interplay and effects on outcome. *Addictive Behaviors, 29*(8), 1665-1672.
116. Read, J. P., Colder, C. R., Merrill, J. E., Ouimette, P., White, J., & Swartout, A. (2012). Trauma and posttraumatic stress symptoms predict alcohol and other drug consequence trajectories in the first year of college. *Journal of Consulting and Clinical Psychology, 80*(3), 426-439.
117. Read, J. P., Wardel, J. D., & Colder, C. R. (2013). Reciprocal associations between ptsd symptoms and alcohol involvement in college: A three-year trait-state-error analysis. *Journal of Abnormal Psychology, 122*(4), 984-997.
118. Read, J. P., Wardell, J. D., Vermont, L. N., Colder, C. R., Ouimette, P., & White, J. (2013). Transition and change: Prospective effects of posttraumatic stress on smoking trajectories in the first year of college. *Health Psychology, 32*(7), 757-767.
119. Reed, P. L., Anthony, J. C., & Breslau, N. (2007). Incidence of drug problems in young adults exposed to trauma and posttraumatic stress disorder: do early life experiences and predispositions matter? *Archives of General Psychiatry, 64*(12), 1435-1442.
120. Richman, J. A., Shinsako, S. A., Rospenda, K. M., Flaherty, J. A., & Freels, S. (2002). Workplace harassment/abuse and alcohol-related outcomes: The mediating role of psychological distress. *Journal of Studies on Alcohol, 63*(4), 412-419.
121. Roberts, T. A., Klein, J. D., & Fisher, S. (2003). Longitudinal effect of intimate partner abuse on high-risk behavior among adolescents. *Archives of Pediatrics and Adolescent Medicine, 157*(9), 875-881.
122. Rogosch, F. A., Oshri, A., & Cicchetti, D. (2010). From child maltreatment to adolescent cannabis abuse and dependence: a developmental cascade model. *Development and Psychopathology, 22*(4), 883-897.
123. Rosario, M., Schrimshaw, E. W., & Hunter, J. (2004). Predictors of substance use over time among gay, lesbian, and bisexual youths: an examination of three hypotheses. *Addictive Behaviors, 29*(8), 1623-1631.
124. Rosen, C. S., Ouimette, P. C., Sheikh, J. I., Gregg, J. A., & Moos, R. H. (2002). Physical and sexual abuse history and addiction treatment outcomes. *Journal of Studies on Alcohol, 63*(6), 683-687.
125. Rospenda, K. M., Fujishiro, K., Shannon, C. A., & Richman, J. A. (2008). Workplace harassment, stress, and drinking behavior over time: Gender differences in a national sample. *Addictive Behaviors, 33*(7), 964-967.
126. Roy, E., Godin, G., Boudreau, J. F., Cote, P. B., Denis, V., Haley, N., . . . Boivin, J. F. (2011). Modeling initiation into drug injection among street youth. *Journal of Drug Education, 41*(2), 119-134.
127. Roy, E., Haley, N., Leclerc, P., Cedras, L., Blais, L., & Boivin, J. F. (2003). Drug Injection among Street Youths in Montreal: Predictors of Initiation. *Journal of Urban Health, 80*(1), 92-105.
128. Ruback, R., Clark, V. A., & Warner, C. (2014). Why are crime victims at risk of being victimized again? Substance use, depression, and offending as mediators of the victimization-revictimization link. *Journal of Interpersonal Violence, 29*(1), 157-185.
129. Russell, D. W., Russell, C. A., Riviere, L. A., Thomas, J. L., Wilk, J. E., & Bliese, P. D. (2014). Changes in alcohol use after traumatic experiences: The impact of combat on Army National Guardsmen. *Drug and Alcohol Dependence, 139*, 47-52.
130. Salomon, A., Bassuk, S. S., & Huntington, N. (2002). The relationship between intimate partner violence and the use of addictive substances in poor and homeless single mothers. *Violence Against Women, 8*(7), 785-815.
131. Schiff, M., Levit, S., & Cohen-Moreno, R. (2010). Childhood sexual abuse, post-traumatic stress disorder, and use of heroin among female clients in Israeli maintenance treatment programs (MMTPs). *Social Work in Health Care, 49*(9), 799-813.
132. Schilling, E. A., Aseltine, R. H., Jr., & Gore, S. (2007). Adverse childhood experiences and mental health in young adults: a longitudinal survey. *BMC Public Health, 7*, 30.
133. Schneider, R., Timko, C., Moos, B., & Moos, R. (2011). Violence victimization, help-seeking, and one- and eight-year outcomes of individuals with alcohol use disorders. *Addiction Research & Theory, 19*(1), 22-31.
134. Shin, S. H., Miller, D. P., & Teicher, M. H. (2013). Exposure to childhood neglect and physical abuse and developmental trajectories of heavy episodic drinking from early adolescence into young adulthood. *Drug and Alcohol Dependence, 127*(1-3), 31-38.
135. Shipherd, J. C., Stafford, J., & Tanner, L. R. (2005). Predicting alcohol and drug abuse in Persian Gulf War veterans: What role do PTSD symptoms play? *Addictive Behaviors, 30*(3), 595-599.
136. Simpson, T. L., Stappenbeck, C. A., Luterek, J. A., Lehavot, K., & Kaysen, D. L. (2014). Drinking motives moderate daily relationships between PTSD symptoms and alcohol use. *Journal of Abnormal Psychology, 123*(1), 237-247.
137. Smith, B., Ryan, M. A., Wingard, D. L., Patterson, T. L., Slymen, D. J., Macera, C. A., & Millennium Cohort Study, T. (2008). Cigarette smoking and military deployment: a prospective evaluation. *American Journal of Preventive Medicine, 35*(6), 539-546.
138. Smith, C. A., Ireland, T. O., Thornberry, T. P., & Elwyn, L. (2008). Childhood maltreatment and antisocial behavior: comparison of self-reported and substantiated maltreatment. *American Journal of Orthopsychiatry, 78*(2), 173-186.
139. Smith, P. H., Homish, G. G., Saddleson, M. L., Kozlowski, L. T., & Giovino, G. A. (2013). Nicotine withdrawal and dependence among smokers with a history of childhood abuse. *Nicotine & tobacco research, 15*(12), 2016-2021.
140. Spears, G. V., Stein, J. A., & Koniak-Griffin Deborah, D. (2010). Latent growth trajectories of substance use among pregnant and parenting adolescents. *Psychology of Addictive Behaviors, 24*(2), 322-332.
141. Sugarman, D. E., Kaufman, J. S., Trucco, E. M., Brown, J. C., & Greenfield, S. F. (2014). Predictors of drinking and functional outcomes for men and women following inpatient alcohol treatment. *American Journal on Addictions, 23*(3), 226-233.
142. Sullivan, T. N., Kung, E. M., & Farrell, A. D. (2004). Relation between witnessing violence and drug use initiation among rural adolescents: parental monitoring and family support as protective factors. *Journal of Clinical Child and Adolescent Psychology, 33*(3), 488-498.
143. Sussman, S., & Dent, C. W. (1999). One-year prospective prediction of marijuana use cessation among youth at continuation high schools. *Addictive Behaviors, 24*(3), 411-417.
144. Sussman, S., & Dent, C. W. (2000). One-year prospective prediction of drug use from stress-related variables. *Substance Use and Misuse, 35*(5), 717-735.
145. Tanaka, M., Wekerle, C., Schmuck, M. L., Paglia-Boak, A., & Team, M. A. P. R. (2011). The linkages among childhood maltreatment, adolescent mental health, and self-compassion in child welfare adolescents. *Child Abuse and Neglect, 35*(10), 887-898.
146. Tarter, R. E., Kirisci, L., Habeych, M., Reynolds, M., & Vanyukov, M. (2004). Neurobehavior disinhibition in childhood predisposes boys to substance use disorder by young adulthood: direct and mediated etiologic pathways. *Drug and Alcohol Dependence, 73*(2), 121-132.
147. Taylor, K. W., & Kliewer, W. (2006). Violence Exposure and Early Adolescent Alcohol Use: An Exploratory Study of Family Risk and Protective Factors. *Journal of Child and Family Studies, 15*(2), 207-221.
148. Testa, M., & Leonard, K. E. (2001). The impact of marital aggression on women's psychological and marital functioning in a newlywed sample. *Journal of Family Violence, 16*(2), 115-130.
149. Testa, M., Livingston, J. A., & Hoffman, J. H. (2007). Does sexual victimization predict subsequent alcohol consumption? A prospective study among a community sample of women. *Addictive Behaviors, 32*(12), 2926-2939.
150. Testa, M., Livingston, J. A., & Leonard, K. E. (2003). Women's substance use and experiences of intimate partner violence: a longitudinal investigation among a community sample. *Addictive Behaviors, 28*(9), 1649-1664.
151. Thaweekoon, T. (2006). *Effects of exposure to community violence on adolescent adjustment problems.* (Dissertation), University of Virginia, Charlottesville, VA.
152. Thompson, M. P., Sims, L., Kingree, J. B., & Windle, M. (2008). Longitudinal associations between problem alcohol use and violent victimization in a national sample of adolescents. *Journal of Adolescent Health, 42*(1), 21-27.
153. Thornberry, T. P., Henry, K. L., Ireland, T. O., & Smith, C. A. (2010). The Causal Impact of Childhood-Limited Maltreatment and Adolescent Maltreatment on Early Adult Adjustment. *Journal of Adolescent Health, 46*(4), 359-365.
154. Topitzes, J., Mersky, J. P., & Reynolds, A. J. (2010). Child maltreatment and adult cigarette smoking: a long-term developmental model. *Journal of Pediatric Psychology, 35*(5), 484-498.
155. Topper, L. R., Castellanos-Ryan, N., Mackie, C., & Conrod, P. J. (2011). Adolescent bullying victimisation and alcohol-related problem behaviour mediated by coping drinking motives over a 12 month period. *Addictive Behaviors, 36*(1-2), 6-13.
156. Turanovic, J. J., & Pratt, T. C. (2013). The consequences of maladaptive coping: Integrating general strain and self-control theories to specify a causal pathway between victimization and offending. *Journal of Quantitative Criminology, 29*(3), 321-345.
157. Ullman, S. E., & Najdowski, C. J. (2009). Revictimization as a moderator of psychosocial risk factors for problem drinking in female sexual assault survivors. *Journal of Studies on Alcohol & Drugs, 70*(1), 41-49.
158. Vos, T., Astbury, J., Piers, L., Magnus, A., Heenan, M., Stanley, L., . . . Webster, K. (2006). Measuring the impact of intimate partner violence on the health of women in Victoria, Australia. *Bulletin of the World Health Organization, 84*(9), 739-744.
159. Waldrop, A. E., & Cohen, B. E. (2014). Trauma exposure predicts alcohol, nicotine, and drug problems beyond the contribution of PTSD and depression in patients with cardiovascular disease: data from the Heart and Soul Study. *American Journal on Addictions, 23*(1), 53-61.
160. Walsh, K., Danielson, C. K., McCauley, J., Hanson, R. F., Smith, D. W., Resnick, H. S., . . . Kilpatrick, D. G. (2012). Longitudinal trajectories of posttraumatic stress disorder symptoms and binge drinking among adolescent girls: the role of sexual victimization. *Journal of Adolescent Health, 50*(1), 54-59.
161. Warren, A. M., Foreman, M. L., Bennett, M. M., Petrey, L. B., Reynolds, M., Patel, S., & Roden-Foreman, K. (2014). Posttraumatic stress disorder following traumatic injury at 6 months: associations with alcohol use and depression. *The Journal of Trauma and Acute Care Surgery, 76*(2), 517-522.
162. Warshaw, M. G., Fierman, E., Pratt, L., Hunt, M., Yonkers, K. A., Massion, A. O., & Keller, M. B. (1993). Quality of life and dissociation in anxiety disorder patients with histories of trauma or PTSD. *American Journal of Psychiatry, 150*(10), 1512-1516.
163. Welch, A. (2011). *Effects of 9/11-related posttraumatic stress disorder on problem alcohol use among World Trade Center Health Registry enrollees.* (Dissertation), The City University of New York, New York.
164. White, H. R., & Widom, C. S. (2008). Three potential mediators of the effects of child abuse and neglect on adulthood substance use among women. *Journal of Studies on Alcohol & Drugs, 69*(3), 337-347.
165. Widom, C. S., & White, H. R. (1997). Problem behaviours in abused and neglected children grown up: Prevalence and co-occurrence of substance abuse, crime and violence. *Criminal Behaviour and Mental Health, 7*(4), 287-310.
166. Widom, C. S., Ireland, T., & Glynn, P. J. (1995). Alcohol abuse in abused and neglected children followed-up: are they at increased risk? *Journal of Studies on Alcohol, 56*(2), 207-217.
167. Widom, C. S., Marmorstein, N. R., & White, H. R. (2006). Childhood victimization and illicit drug use in middle adulthood. *Psychology of Addictive Behaviors, 20*(4), 394-403.
168. Widom, C. S., Weiler, B. L., & Cottler, L. B. (1999). Childhood victimization and drug abuse: a comparison of prospective and retrospective findings. *Journal of Consulting and Clinical Psychology, 67*(6), 867-880.
169. Widom, C. S., White, H. R., Czaja, S. J., & Marmorstein, N. R. (2007). Long-term effects of child abuse and neglect on alcohol use and excessive drinking in middle adulthood. *Journal of Studies on Alcohol & Drugs, 68*(3), 317-326.
170. Wilson, H. W., & Widom, C. S. (2010). Predictors of drug-use patterns in maltreated children and matched controls followed up into middle adulthood. *Journal of Studies on Alcohol & Drugs, 71*(6), 801-809.
171. Wilson, H. W., & Widom, C. S. (2010). The role of youth problem behaviors in the path from child abuse and neglect to prostitution: A prospective examination. *Journal of Research on Adolescence, 20*(1), 210-236.
172. Wilson, S. M., Dedert, E. A., Dennis, P. A., Dennis, M. F., Calhoun, P. S., Kirby, A. C., & Beckham, J. C. (2014). Do ethnicity and gender moderate the influence of posttraumatic stress disorder on time to smoking lapse? *Addictive Behaviors, 39*(7), 1163-1167.
173. Wislar, J. S., Richman, J. A., Fendrich, M., & Flaherty, J. A. (2002). Sexual harassment, generalized workplace abuse and drinking outcomes: The role of personality vulnerability. *Journal of Drug Issues, 32*(4), 1071-1088.
174. Wolitzky-Taylor, K., Bobova, L., Zinbarg, R. E., Mineka, S., & Craske, M. G. (2012). Longitudinal investigation of the impact of anxiety and mood disorders in adolescence on subsequent substance use disorder onset and vice versa. *Addictive Behaviors, 37*(8), 982-985.
175. Wollard, C. A. (2003). *Psychosocial predictors and mediators of drug use and eating problems in women: A community sample longitudinal study.* (Dissertation), University of Southern California, Los Angeles, CA
176. Wright, E. M., Fagan, A. A., & Pinchevsky, G. M. (2013). The effects of exposure to violence and victimization across life domains on adolescent substance use. *Child Abuse and Neglect, 37*(11), 899-909.
177. Wright, K. M., Foran, H. M., Wood, M. D., Eckford, R. D., & McGurk, D. (2012). Alcohol problems, aggression, and other externalizing behaviors after return from deployment: understanding the role of combat exposure, internalizing symptoms, and social environment. *Journal of Clinical Psychology, 68*(7), 782-800.
178. Wu, P., Bird, H. R., Liu, X., Duarte, C. S., Fuller, C., Fan, B., . . . Canino, G. J. (2010). Trauma, posttraumatic stress symptoms, and alcohol-use initiation in children. *Journal of Studies on Alcohol & Drugs, 71*(3), 326-334.
179. Yang, C., German, D., Webster, D., & Latkin, C. (2011). Experiencing violence as a predictor of drug use relapse among former drug users in Baltimore, Maryland. *Journal of Urban Health, 88*(6), 1044-1051.
180. Zlotnick, C., Johnson, D. M., & Kohn, R. (2006). Intimate Partner Violence and Long-Term Psychosocial Functioning in a National Sample of American Women. *Journal of Interpersonal Violence, 21*(2), 262-275.
181. Zweig, J. M., Yahner, J., & Rossman, S. B. (2012). Does recent physical and sexual victimization affect further substance use for adult drug-involved offenders? *Journal of Interpersonal Violence, 27*(12), 2348-2372.
